# Role of Hsp70 ATPase Domain Intrinsic Dynamics and Sequence Evolution in Enabling its Functional Interactions with NEFs

**DOI:** 10.1371/journal.pcbi.1000931

**Published:** 2010-09-16

**Authors:** Ying Liu, Lila M. Gierasch, Ivet Bahar

**Affiliations:** 1 Department of Computational and Systems Biology, School of Medicine, University of Pittsburgh, Pittsburgh, Pennsylvania, United States of America; 2 Department of Biochemistry and Molecular Biology, and Department of Chemistry, University of Massachusetts Amherst, Amherst, Massachusetts, United States of America; Columbia University, United States of America

## Abstract

Catalysis of ADP-ATP exchange by nucleotide exchange factors (NEFs) is central to the activity of Hsp70 molecular chaperones. Yet, the mechanism of interaction of this family of chaperones with NEFs is not well understood in the context of the sequence evolution and structural dynamics of Hsp70 ATPase domains. We studied the interactions of Hsp70 ATPase domains with four different NEFs on the basis of the evolutionary trace and co-evolution of the ATPase domain sequence, combined with elastic network modeling of the collective dynamics of the complexes. Our study reveals a subtle balance between the intrinsic (to the ATPase domain) and specific (to interactions with NEFs) mechanisms shared by the four complexes. Two classes of key residues are distinguished in the Hsp70 ATPase domain: (i) highly conserved residues, involved in nucleotide binding, which mediate, via a global hinge-bending, the ATPase domain opening irrespective of NEF binding, and (ii) not-conserved but co-evolved and highly mobile residues, engaged in specific interactions with NEFs (e.g., N57, R258, R262, E283, D285). The observed interplay between these respective intrinsic (pre-existing, structure-encoded) and specific (co-evolved, sequence-dependent) interactions provides us with insights into the allosteric dynamics and functional evolution of the modular Hsp70 ATPase domain.

## Author Summary

The heat shock protein 70 (Hsp70) serves as a housekeeper in the cell, assisting in the correct folding, trafficking, and degradation of many proteins. The ATPase domain is the control unit of this molecular machine and its efficient functioning requires interactions with co-chaperones, including, in particular, the nucleotide exchange factors (NEFs). We examined the molecular motions of the ATPase domain in both NEF-bound and -unbound forms. We found that the NEF-binding surface enjoys large global movements prior to NEF binding, which presumably facilitates NEF recognition and binding. NEF binding stabilizes the ATPase domain in an open form and thereby facilitates the nucleotide exchange step of the chaperone cycle. A series of highly correlated amino acids were distinguished at the NEF-binding sites of the Hsp70 ATPase domain, which highlights the adaptability of the ATPase domain, both structurally and sequentially, to recognize NEFs. In contrast, the nucleotide-binding residues are tightly held near a global hinge center and are highly conserved. The contrasting properties of these two groups of residues point to an evolutionarily optimized balance between conserved/constrained and co-evolved/mobile amino acids, which enables the functional interactions of the modular Hps70 ATPase domains with NEFs.

## Introduction

Many proteins are molecular machines. They function because their three-dimensional structure allows them to undergo cooperative changes in conformation that maintain the native fold while enabling their biological functions. The changes have been pointed out to be structure-encoded, intrinsically accessible to proteins, as can be deduced from simple physics-based approaches [1]. Yet, amino acid specificity is another important property that selectively mediates the interactions with specific partners and ligands [2]. Overall, a subtle balance exists between structure-encoded mechanical properties and sequence-encoded specific properties, and this balance must be evolutionarily optimized to achieve precise functioning.

The interplay between these two effects becomes particularly important in the case of a number of proteins or domains that play a modular role in a variety of biomolecular interactions. The ATPase domain (also called nucleotide-binding domain) of the Hsp70 family of proteins is a typical example. This domain plays a critical role in regulating the activities of these molecular chaperones, which, in turn, promote accurate folding, and prevent unwanted aggregation by either unfolding and refolding misfolded proteins or regulating their intracellular trafficking to the protein degradation machinery [3]–[5].

Chaperones of the Hsp70 family contain two domains: the N-terminal ATPase domain and the C-terminal substrate-binding domain (SBD), which regulate each other's activity via allosteric effects. ATP hydrolysis at the ATPase domain increases the substrate-binding affinity of the SBD, thus lowering the substrate exchange rate; on the other hand, the dissociation of the ADP produced upon ATP hydrolysis and its replacement by a new ATP trigger the release of substrate by the SBD, and therefore enhance the substrate exchange rate [3]. Regulation of substrate-binding affinity by the ATPase domain forms the basis of the chaperone activity of Hsp70s [6], [7].

The precise functioning of the Hsp70 ATPase domain involves an interaction with two families of co-factors, also called co-chaperones: the J-domain proteins that catalyze ATP hydrolysis [8], and the nucleotide exchange factors (NEFs) that assist in the replacement of ADP with ATP, by significantly increasing the ADP dissociation rate [9]. A molecular understanding of Hsp70 function requires a systemic analysis of the structural basis and mechanism of interaction with these co-chaperones. Here we focus on the interaction of their ATPase domain with NEFs.

**Figure 1:**
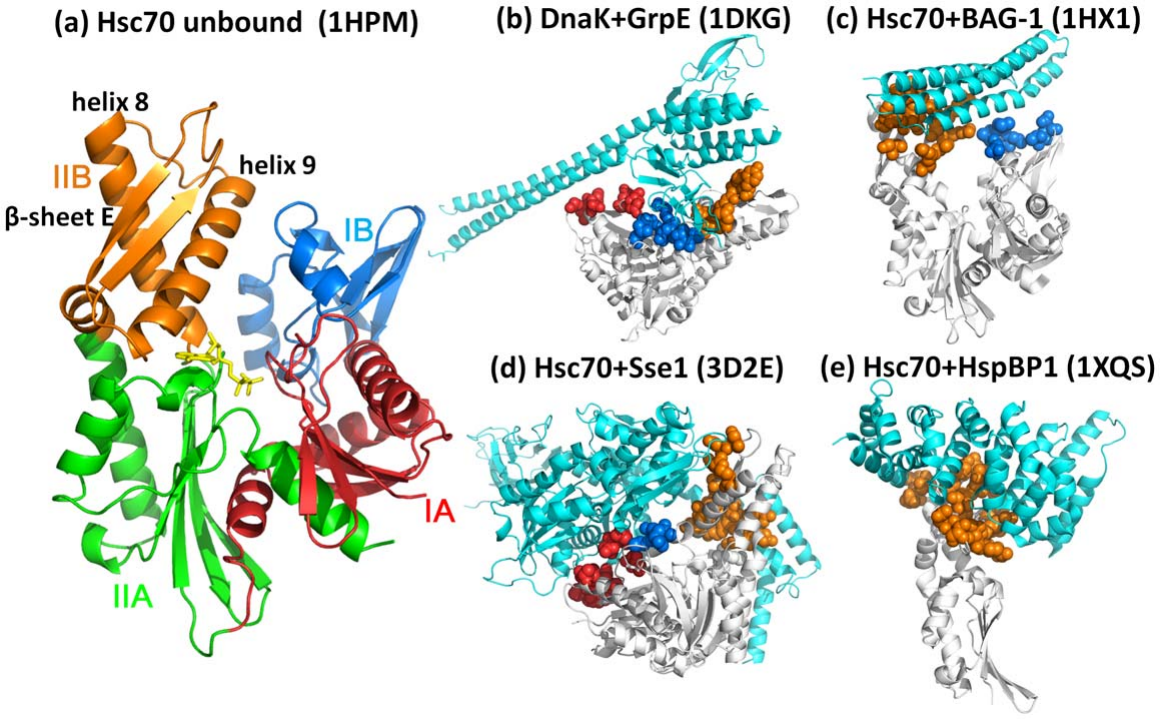
Structure of Hsp70 ATPase domain and its complexes with different nucleotide exchange factors (NEFs). **(a) ATPase domain structure colored by subdomains:** IA (red; residues 1–39 and 116–188), IB (blue; residues 40–115), IIA (green; residues 189–228 and 307-C-terminus) and IIB (orange; residues 229–306). Several subdomain IIB residues are involved in NEF recognition and binding, including residues at the C-terminal part of helix 8 (G230-H249), the helix 9 (K257-S275), and the β-sheet E (strands Q279-I284 and F293-T298 connected by a long exposed loop). Residue identifications and secondary structure nomenclature are based on the PDB entry 1HPM. In yellow stick representation is a bound ADP. **(b–e) Interactions with four different NEFs.** (**b**) DnaK ATPase fragment from *E. coli* complexed with GrpE, (**c**) bovine Hsc70 complexed with BAG-1, (**d**) human Hsc70 with Sse1, and (**e**) human Hsc70 with HspBP1. In each case the NEF is colored cyan, ATPase fragment white, and interface residues, shown in space-filling representation, are colored according to their subdomain locations. See **Table S1** and Table **S2**, and the *Materials and Methods* for more information on the examined complexes, and the identity of NEF-recognition residues in each case. All ribbon diagrams are created using PyMol (http://www.pymol.org).

The Hsp70 ATPase domain is composed of four subdomains: IA and IB in lobe I, and, IIA and IIB in lobe II (**Figure 1a**). ATP binds the central cleft between the two lobes at the interface between subdomains IIA and IIB such that the geometric and energetic effects of its binding and hydrolysis are efficiently transmitted throughout the ATPase domain.

To date, four classes of NEFs have been identified: GrpE in prokaryotes [10], and BAG-1 [11], HspBP1 [12] and Hsp110 [13] in eukaryotes. Their diverse three-dimensional structures (**Figure 1b–e**) exhibit a variety of binding geometries and interfacial interactions with the Hsp70 ATPase domain. In the present study, we examine these interactions, using sequence-, structure- and dynamics-based computations and identify their shared features. Our analysis provides insights into the generic and specific aspects of ATPase domain-NEF interactions, as well as the molecular machinery and sequence design principles of this highly versatile module, the Hsp70 ATPase domain, thus reconciling robust structure-encoded cooperative dynamics properties and highly correlated amino acid changes that enable specific recognition.

## Materials and Methods

### Multiple sequence alignment of Hsp70 ATPase domain

We began with 4839 sequences retrieved from the Pfam database 22.0 [14] for the Hsp70 family of molecular chaperones (Pfam id: PF00012). We refined the generated multiple sequence alignment (MSA) by using the consensus sequence of the ATPase domain (380 residues) in the bovine cytosolic homolog of Hsp70 [15]. The refinement consists of three steps: (i) iterative implementation of Smith-Waterman algorithm (SW) for pairwise alignment [16] using our consensus sequence, and elimination of those sequences below a threshold SW score (or less than 40% sequence identity; see details in Supplementary Material (*SM*) **Figure S1** and **Text S1**) to retrieve the closest orthologs to human (Hsc70) and bacterial (DnaK) chaperones; (ii) deletion of MSA columns that correspond to insertions with respect to the consensus sequence, and (iii) removal of the sequences containing more than 10 gaps. These three steps resulted in a MSA of 1627 sequences with *N* = 380 columns (corresponding to residues 6 to 385 in Hsc70 ATPase domain), which has been subjected to evolutionary trace (ET) and mutual information (MI) analyses for detecting residue conservation and co-evolution patterns, respectively.

### Structural data

We retrieved from the Protein Data Bank (PDB) [17] structural data for HSP70 ATPase domains complexed with GrpE (PDB id: 1DKG [10]), BAG-1 (PDB id: 1HX1 [18]), HspBP1 (PDB id: 1XQS [19]), and Sse1 (Hsp110, PDB id: 3D2E [20]), shown in **Figure 1b–e**. Additionally, the structure of the above mentioned bovine Hsc70 ATPase domain resolved at 1.7 Å resolution (PDB id: 1HPM [15]) was used for the unbound form, and the PDB structure 1S3X [21] of the human Hsp70 served as a template to reconstruct the lobe I missing in the complex with HspBP1 using the method described in the *SM* **Figure S2** and **Text S2**.

### Elastic network models and comparison of global modes with experimental data

We performed Gaussian Network Model (GNM) [22], [23] and anisotropic network model (ANM) [24], [25] analyses for elucidating the equilibrium dynamics of Hsp70 ATPase domain both in the unbound form and in the complexes with different NEFs, including the reconstructed complex with HspBP1. Details on the methods can be found in our previous work [22]–[26]. Mainly, knowledge of the distribution of inter-residue contacts in the native structure permits us to construct the Kirchhoff (GNM) and Hessian (ANM) matrices, which, upon eigenvalue decomposition, yield information on the collective modes spectra.

**Figure 2:**
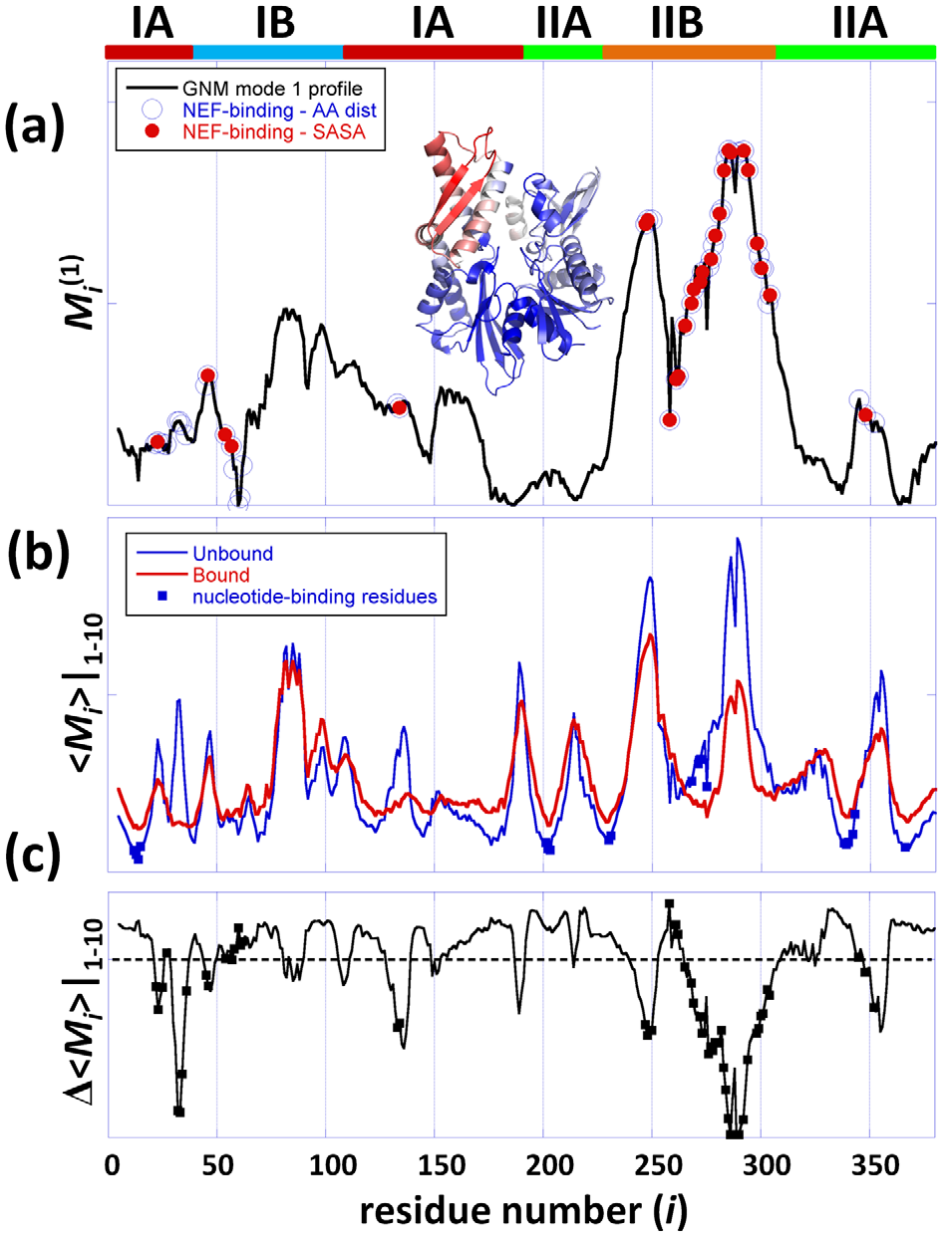
Intrinsic dynamics of the Hsp70 ATPase domain: high mobility of NEF-recognition sites in contrast to restricted mobility of nucleotide-binding residues. (**a**) Distribution of residue mobilities *M_i_* ^(1)^ in the global mode of motion calculated for the unbound Hsp70 ATPase domain. The horizontal bars on the upper abscissa indicate the ranges of the four subdomains IA, IB, IIA and IIB, colored as in **Figure 1a**. Subdomain IIB is distinguished by its enhanced mobility, with peaks at two regions: the C-terminal part of helix 8, and the β-hairpin loop. NEF-binding residues are indicated by the blue open circles (based on atom-atom distances) and red filled circles (based on ΔSASA). The diagram in the inset is color-coded to illustrate the global mobility profile (red: most mobile, blue: most rigid). (**b**) Weighted-average mobility profiles based on top-ranking ten GNM modes of motion, calculated using Equation 1 for the unbound ATPase domain (blue) and for the NEF-bound structures (red), averaged over three mammalian complexes (**Table S1**). Nucleotide-binding residues (G12-Y15, G201-G203, G230, E231, E268, K271, R272, S275, G338- S340, R342, I343, D366) are indicated by filled squares. (**c**) Change in mobility between bound and unbound ATPase domain, obtained by taking the difference of two curves shown in panel **b**. The dashed line corresponds to the zero level. NEF-binding residues are marked by filled squares.

We focused on the low frequency modes, also called *global* modes, as the major determinant of functional movements. In the GNM, each mode *k* is represented by an *N*-dimensional eigenvector, ***u*** ^(*k*)^, and eigenvalue *λ_k_*, describing the mode shape and frequency (squared), respectively. The *i* ^th^ element, [***u*** ^(*k*)^]*_i_*, of ***u*** ^(*k*)^ describes the displacement of residue *i* along the *k* ^th^ mode axis; the plot of [***u*** ^(*k*)^]*_i_* ^2^ as a function of residue index *i* defines the *mobility profile M_i_* ^(*k*)^ in mode *k*. See for example, the mobility profile *M_i_* ^(*1*)^ for the first (lowest frequency) mode accessible to Hsp70 ATPase domain in **Figure 2a**. By definition, eigenvectors are normalized, i.e., the mobility profile also represents the normalized distribution of square displacements in mode *k*. The reciprocal *λ_k_* ^−1^ serves as the weight of mode *k*, such that the slower modes, also called *softer modes*, make larger contributions to observed dynamics. The mobility of residue *i* driven by a subset of *m* soft modes is found from the weighted average (**Figure 2b**)
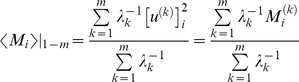
(1)


The modes predicted by the ANM for the ATPase domain both in NEF-bound and –free forms were compared to the experimentally measured changes in structure (designated by a 3*N*-dimensional deformation vector ***d***) using two metrics: (i) the correlation cosine (|***v*** ^(*k*)^·***d***|/|***d***|) between the *k* ^th^ ANM eigenvector ***v*** ^(*k*)^ (*k* = 1,…, 3*N*-6) and ***d***, and (ii) the cumulative overlap achieved by the *m* softest modes [26],
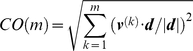
(2)The deformation ***d***, is obtained by superposing the known NEF-bound and -free structures of ATPase domain and evaluating the differences in the C^α^-coordinates. Kabsch's algorithm [27] is used for optimal superposition that eliminates rigid-body translations and rotations.

### Evolutionary Trace (ET) method

**Figure 3:**
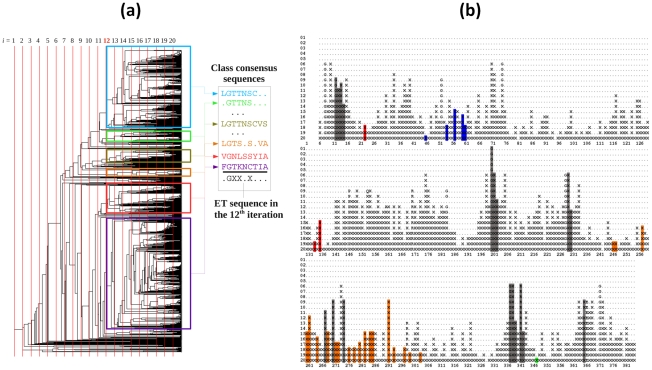
Schematic description of ET calculations for Hsp70 family. (**a**) The phylogenetic tree is constructed using the ET server [30] and the set of 1627 ATPase domain sequences retrieved from PFAM database for the Hsp70 family. Each vertical line corresponds to a given distance threshold. The boxes in different colors refer to the partitions obtained at the 12th distance threshold (also called level). Each box yields a different consensus sequence. The class consensus sequence for each partitioning level is then identified, as illustrated. Dots therein refer to positions that are sequentially variable between the members of the class. The ET sequence for the particular level is determined by assigning letter code X to all positions that are conserved *within* classes, but not conserved *across* classes. Those amino acids conserved across classes are indicated by their single letter code (e.g., glycine G in the illustrated ET sequence). (**b**) Results are shown for a 20-level partitioning of the phylogenetic tree. Peaks indicate the most conserved residues (among the 380 amino acids represented in each sequence), with their conservation level (or ET rank) indicated by the row numbers on the left. The columns highlighted in gray correspond to nucleotide binding residues. Those corresponding to the NEF binding residues are colored by the subdomains to which they belong (see **Figure 1a**). A high-resolution version of this figure can be seen in the **Figure S3** of the *SM*.

The ET method [28] identifies conserved residues using the MSA-derived phylogenetic tree for a given family. The application of the procedure to the Hsp70 family of chaperones is outlined in **Figure 3**, and details can be found in previous work [28], [29]. In summary, the method consists of three steps: (i) the phylogenetic tree is partitioned into multiple levels [30] as indicated by the vertical bars in **Figure 3a**; (ii) at each level, sequences are grouped into classes, each being characterized by a “class consensus sequence”. The consensus sequences are cross-examined to identify fully conserved (across classes) and class-specific or *trace* residues (conserved within classes but not across classes). The *ET sequence* for the particular level lists the fully conserved residues by their single-letter code, the trace residues by the symbol ‘X’, and the remaining residues as blank; and (iii) The ET sequences generated at each level are organized in rows (**Figure 3b**). An ET rank (*leftmost column*) is assigned to each residue. A fully conserved residue is assigned the highest rank (rank of 1). In the present case, Gly201 is the only residue with ET rank 1, i.e., it is fully conserved among the set of 1627 sequences (see *SM* **Figure S3** for a larger version of this panel).

The conservation of a given residue in all subfamilies is a very strict condition when large sets of aligned sequences are considered. This limitation restricted the previous applications of the ET method to MSA of 100 and 200 sequences [31]. To adapt the ET method and its variations [32]–[34] to our dataset of >1,600 sequences, we relaxed the condition for defining an ET residue from conservation across “all” members in a given level to “90%”of members, and we allowed for gaps [34].

### Mutual Information (MI)

The ET method identifies conserved residues, but does not provide information on co-evolutionary relations between residues. Co-evolving residues are usually indicative of structural or functional constraints [35]–[38]. We adopted the MI content as a measure of the degree of intra-molecular co-evolution between residues in the Hsp70 ATPase domain [37], [39]–[41]. In this method, each of the *N* columns of the MSA is considered as a discrete random variable that takes on one of the 20 amino-acid types, or an insertion (gap, as the 21^st^ type), with some probability. The MI associated with the *i* ^th^ and *j* ^th^ sequence positions is defined as an *N*×*N* matrix (for a MSA of *N* columns) of the form
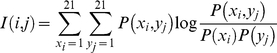
(3)where *P*(*x_i_*, *y_j_*) is the joint probability of observing amino acid types *x* and *y* at the respective sequence positions *i*, and *j*; *P*(*x_i_*) is the marginal/singlet probability of amino acid of type *x* at the *i^th^* position. *I*(*i*, *j*) varies in the range [0, *I*_max_], where the lower and upper limits correspond to fully uncorrelated and most correlated pairs of residues.

## Results

Here is a brief summary of the approach and rationale. First, we examine the structural properties of known Hsp70 ATPase domain-NEF complexes from different organisms to identify the interfacial residues. Second, we analyze the intrinsic (structure-encoded) dynamics of the ATPase domain using the GNM, with an eye on the dynamic characteristics of the NEF-binding residues, on the one hand, and ATP/ADP-binding residues, on the other. A clear difference emerges between these two groups of functional residues: the former is distinguished by enhanced mobility in the softest modes while the latter is severely restricted. Third, calculations repeated with NEF-bound ATPase domains reveal how the open form of the ATPase domain is stabilized in order to facilitate ADP release, which is enabled by the intrinsic mobility of the NEF-binding regions. Nucleotide-binding sites, on the other hand, are shown to maintain their generic structure and dynamics irrespective of NEF binding, pointing to the robustness of the ATP-regulation by the ATPase domain. Fourth, detailed sequence analysis of Hsp70 family members reveals the distinctive sequence properties of the two regions: NEF-binding sites exhibit highly correlated mutations, consistent with the recognition of specific NEFs. Nucleotide-binding sites on the other hand, are almost fully conserved. In a sense, sequence variability is accompanied by conformation variability and *vice versa*.

Overall, Hsp70 ATPase domains appear to have been evolutionarily optimized to acquire a dual character: functional variability accompanied by structural variability at the co-chaperone binding sites and conservation/robustness both in terms of sequence and structural dynamics at the nucleotide-binding sites. This dual character is proposed to be essential for adapting to interactions with different co-factors while maintaining ATPase activity.

### Structural dynamics

#### All NEFs are in contact with subdomain IIB on the ATPase domain

We examined the interface between the Hsp70 ATPase domain and the corresponding NEF in four structurally resolved complexes: with GrpE, BAG-1, HspBP1 or Sse1. Despite their structural differences, all four NEFs make contacts with subdomain IIB (**Figure 1b–e**). Subdomain IIB regions involved in contacts with NEFs include the α-helices 8 and 9, the double-stranded β-sheet E, and the loop connecting the two strands (**Figure 1a**). The NEF-contacting surface also includes small regions in subdomains IA and IB, but rarely IIA. The complete lists of Hsp70 ATPase domain residues that make contacts with each of the four NEFs may be seen in the *SM* **Table S1** and Table **S2**. **Table S1** is based on atom-atom interactions closer than 4Å separation. **Table S2** is based on the change in solvent-accessible surface areas, Δ(SASA), induced upon NEF binding. The entries in **Table S2** form a subset of those in **Table S1**, thus helping consolidate the identity of the NEF-binding residues on the ATPase domain. We note in particular Asn57, Arg258, Arg261, Arg262 and Tyr134 shared by both mammalian and bacterial chaperones in their NEF binding activity. **Table S2** also lists the interfacial NEF residues for each case, which draws attention to the abundance of salt bridges at the mammalian Hsc70/NEF interfaces. In contrast, DnaK-GrpE contacts are predominantly hydrophobic, consistent with previous observations [18].

#### Intrinsic dynamics of the Hsp70 ATPase domain: High mobility of subdomain IIB

Results from the GNM analysis of Hsp70 ATPase domain dynamics are presented in **Figure 2**. Panel **a** displays the mobility profile *M_i_* ^(1)^ in the lowest frequency (global) mode of motion intrinsically favored by the overall ATPase domain architecture (calculated for the unbound Hsc70 ATPase domain (1HPM, [15]). Subdomain IIB is distinguished by its enhanced mobility (see also the color-coded diagram in the inset of **Figure 2a**). Interestingly, this region also contains the primary contact surface with NEFs. The symbols on the curve indicate the sequence positions of NEF-contacting residues identified using two methods: atom-atom contacts (blue open circles) and Δ(SASA) (red filled circles). In particular, Glu283, Asp285, Ser286, Asp292 and Tyr294 at β-sheet E loop form the highest peak in the mobility profile, succeeded by Arg247-Lys248 on helix 8 C-terminus, suggesting that these residues play a role in NEF recognition.

Thus, the subdomain that makes the majority of the contacts with the NEFs (i.e., subdomain IIB) is the one favored by the Hsp70 ATPase domain architecture to enjoy the largest mobility in the most cooperative mode of motion accessible to the ATPase domain. We also note, among NEF-contacting residues, a few exhibiting more restricted mobilities, located at the interface between subdomains IB and IIB, in particular. The tendency of biomolecules to involve their most mobile regions (peaks in the softest modes) in ligand recognition appears to be a design property noted in other applications; the ATPase domain subdomain IIB conforms to this rule. Its intrinsic mobility or conformational adaptability presumably allows for optimal interaction with the bound NEFs. On the other hand, final stabilization of a ‘bound’ conformer and communication of the conformational change locked upon substrate binding to other functional sites (e.g., nucleotide-binding site, in this case), may require the involvement of spatially constrained regions near the binding site [42]. The binding site thus tends to exhibit a dual character, comprising both highly mobile residues that easily reconfigure for optimal binding and spatially constrained residues that efficiently communicate the structural change (from unbound to bound form) to other functional parts of the molecule [43], [44]. NEF-binding residues His23 in subdomain IA, N57, Ala60 and Met61 in subdomain IB, and Arg258 and Arg261 in subdomain IIB presumably assume this allosteric communication role, as will be further clarified below.

#### The Hsp70 nucleotide-binding site coincides with a rotationally flexible but spatially immobile global hinge region

The Hsp70 nucleotide-binding residues, on the other hand, represent a completely different type of behavior. These residues, indicated by the blue squares in **Figure 2b** (and listed in the caption), occupy regions that are severely constrained in the low frequency modes, i.e., they undergo minimal, if any, displacements in the collective movements of the entire domain. They participate in precisely tuned interactions at the global hinge region that mediates the concerted movements of the subdomains, and as such they need to remain in their key mechanical positions. Their lack of mobility, or displacement/translation in space, does not imply lack of rotational flexibility, however. On the contrary, in the same way as hinges operate, these residues are fixed in space, but have highly rotatable bonds that allow for the relative motions of the adjoining subdomains. Not surprisingly, this set has an abundance of glycines (G12, G201, G202, G203, G230, G338 and G339). The hinge bending role of these residues is critical to enabling the opening of the nucleotide binding pocket in response to NEF binding.

We also note among nucleotide-binding residues three charged residues, K271, R272 and R342, which were distinguished by their ‘central’ role in mediating the communication between the nucleotide-binding site and the other parts of the Hsp70 ATPase domain [45]. Their central role was deduced from the small-world network approach introduced by del Sol and coworkers [46], [47].

The co-localization of chemically active sites with the global hinge region is another design feature consistent with previous observations reported for catalytic sites of enzymes [42].

It will be shown below that the NEF-contacting and nucleotide-binding residues form two groups fundamentally different in terms of their evolutionary properties, in addition to their contrasting (highly mobile *vs* highly constrained) dynamics in the global modes intrinsically accessible to the Hsp70 ATPase domain.

#### NEF binding suppresses the motions of subdomain IIB and stabilizes an open conformer

**Figure 2b** compares the mobility profiles obtained for Hsp70 ATPase domains in the NEF-free form (blue curve) with the average profile exhibited by three NEF-bound structures (with mammalian homologues 1HX1, 1XQS and 3D2E in **Table S1**). For clarity, the average over these three cases (red curve) is displayed in **Figure 2b**, and the individual curves for each complex may be seen in the *SM* **Figure S4**. In each case, the ten top (lowest frequency) modes are used to display the weight-averaged square displacements, which provide an accurate representation of the overall collective dynamics. The results indicate that the NEF-bound form of the Hsp70 ATPase domain closely maintains the intrinsic dynamics accessible to its unbound form, i.e., the loci of peaks and minima remain practically unchanged; however, binding of a NEF alters the relative (quantitative) distribution of mobilities: in particular, a reduction is observed in the mobility of subdomain IIB. As can be seen in more details for each of the four complexes in the *SM* **Figure S4**, the peak around the β-hairpin loop (residues 285–292) in subdomain IIB is almost completely depressed in the case of Sse1 and BAG-1, while GrpE and HspBP1 binding suppresses the mobility of the C-terminal end of helix 8 in the same subdomain.

**Figure 2c** shows the change in the mobility profile of the Hsp70 ATPase domain upon NEF binding. In addition to suppressed motions at the β-hairpin, we also observe a drop in mobility at a number of NEF-contacting residues in subdomain IA (e.g., D32-G34). Notably, while NEF-binding residues on the ATPase domain experience reduced mobility upon NEF binding, the NEFs themselves enjoy large conformational freedom, as illustrated in the *SM* **Figure S5**, as if their global fluctuations are conferred by the dissipation of those in the ATPase domain.

By and large, all these observations support a common mechanism shared by all NEFs: they bind the most mobile subdomain of the Hsp70 ATPase domain - subdomain IIB, and in essence ‘lock’ the ATPase domain in a fixed conformation. This newly stabilized conformation is the ‘open’ form of the ATPase domain, as will be elaborated below.

#### Induced vs. intrinsic dynamics?

**Figure 4:**
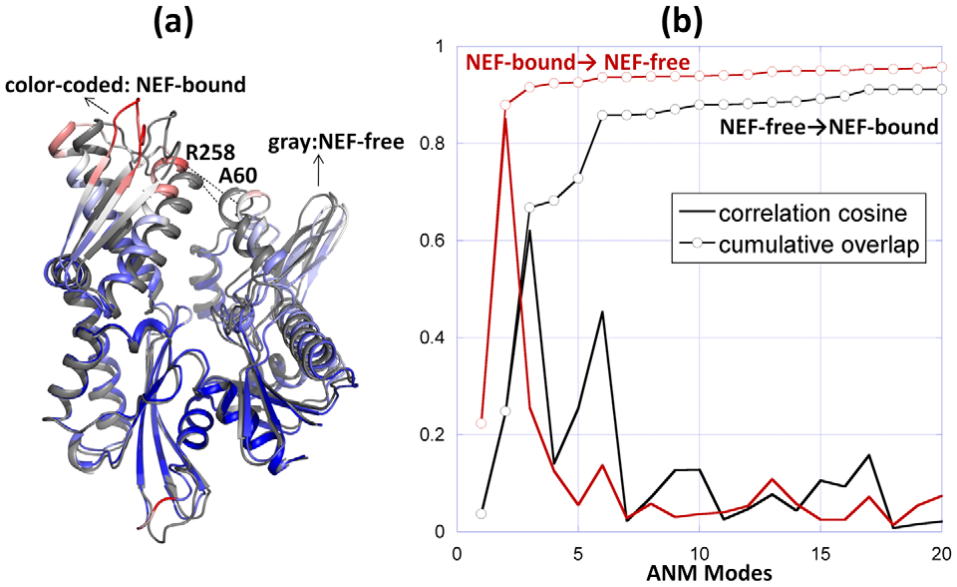
Comparison of experimentally observed and computationally predicted structural changes in the Hsp70 ATPase domain. Experimental changes are illustrated for BAG-1-bound and free forms of the bovine Hsp70 ATPase domain (respective PDB Ids: 1HX1 and 1HPM). Computational results are obtained by the ANM applied to the respective two structures. (**a**) Structural alignment of NEF-bound and unbound ATPase fragments. The unbound ATPase fragment (1HPM) is colored gray. The NEF-bound ATPase fragment (1HX1) is color-coded according to its extent of deformation with respect to the unbound ATPase, the regions showing the largest deformation being colored red, and those unchanged, blue. The distance between Ala60 and Arg258 C^α^-atoms is 5.0 Å in the closed form and 10.9Å in the open form. Panel **b** displays the results for the unbound (black) and BAG-1-bound (red) ATPase domain. The solid curve represents the correlation cosine between the experimentally observed deformation vector ***d*** and the ANM modes 1–20 accessible to the ATPase domain (either NEF-bound or -free). The curve with circles describes the cumulative overlap (Equation 2). A subset of 6 slow modes accessible to the unbound form ensures the passage to the NEF-bound conformer with an overlap of 0.86. The NEF-bound form exhibits an even stronger potential to be reconfigured back to its closed form, consistent with the preferred conformation of the ATPase domain in the absence of NEF binding: top ranking two modes yield a cumulative overlap of 0.88 with the experimental deformation ***d***.

Comparison of the NEF-bound and –unbound structures of the Hsp70 ATPase domain shows that the distance between the subdomains IIB and IB is larger in the NEF-bound form. **Figure 4a** illustrates this ‘opening’ for BAG-1-bound ATPase domain. In this complex, subdomain IIB undergoes a rotation of 14° with respect to the rest of the structure [18], and in Sse1-bound ATPase domain, subdomain IIB is observed to rotate 27° sideways. The stabilization of an open conformer is a common feature in all NEF-bound structures, although they exhibit slight variations in the detailed geometry of the accompanying conformational changes [18]–[20], [48]. By stabilizing the open form, NEFs assist in increasing the nucleotide exchange rate and communication with the SBD.

The observed conformational change of the Hsp70 ATPase domain may be explained by three possible scenarios: (i) *induced* upon NEF binding, (ii) a *pre-existing* equilibrium/path where the open form is already sampled, or can be readily reached via a soft mode, by the NEF-free ATPase domain, (iii) pre-existing equilibrium/path followed by induced fit. The former would be NEF-specific; the latter, would be intrinsic to the ATPase domain; and the third is an intermediate behavior, i.e. the original recognition requires the pre-disposition of the suitable ‘binding’ conformation (pre-existing equilibrium); and binding of NEF induces further rearrangements to optimize the intermolecular interactions. For a more extensive discussion see for example our previous work [49]. Clearly, in the case of intrinsically disordered proteins, folding upon binding is a common phenomena [50], [51], in line with an induced fit. On the other hand, structural adaptability to increase substrate specificity would be explained by scenarios (ii) or (iii) [2].

In order to examine quantitatively to what extent the observed reconfiguration is an intrinsic property of the Hsp70 ATPase domain (as opposed to a property induced by NEF), we focused on the softest motions predicted by the ANM. The black curve in **Figure 4b** displays the correlation cosine between the ANM modes (***v*** ^(*k*)^, *k* = 1–20) predicted to be intrinsically accessible to the unbound ATPase domain and the experimentally observed deformation (a 3*N*-dimensional vector ***d***; see *Materials and Methods*) between the open and closed forms of the ATPase domain. Note that the complete space of equilibrium motions comprises a collection of 3*N*-6 modes in the ANM, and by definition these form an orthonormal basis vector such that a cumulative overlap *CO*(*3N-6*) = 1 is obtained by adding up all modes' contributions (see Equation 2). In the absence of correlations between the predicted modes and the experimentally observed changes, *i.e.*, if the modes were completely random, their correlation cosine with ***d*** would therefore be (3*N*-6)^−1/2^ = 0.029, using *N* = 380. In contrast, a single mode alone (*k* = 3) is found here to exhibit a correlation cosine of 0.62 with the observed deformation, and the cumulative overlap reaches 86% by moving along 6 modes only (black curve with circles **in** **Figure 4b**). This result suggests that NEF binding exploits to a large extent the reconfiguration accessible to the Hsp70 ATPase domain via this particular mode to drive the transition of the Hsp70 ATPase domain from its closed (NEF-free) state to an open (NEF-bound) state. Selection from a pre-existing path appears to be the dominant mechanism, although there is a minor contribution from higher modes selected via induced fit mechanism, in support of scenario (iii).

We further explored the transition between the open and closed forms of the Hsp70 ATPase domain by examining the reverse process, i.e., we examined the ability of the *open* form of the ATPase domain to restore its conformation back to the closed form in the absence of a NEF (red curves in **Figure 4b**). The results show that the intrinsic tendency to go back to the closed form is even stronger (than the tendency to open up). In fact, the 2^nd^ softest mode in this case exhibits a correlation cosine of 0.85 alone with the experimentally observed deformation ***d***. Therefore, the movement along this single mode coordinate is practically sufficient to restore a significant portion of the conformational perturbation selectively stabilized by NEF. Calculations performed for different NEF-bound forms exhibited similar features. See for example those obtained for Sse1-bound ATPase domain in the *SM* **Figure S6**. We conclude that the restoration of the NEF-free conformation after the dissociation of NEF is an intrinsic change almost exclusively favored by pre-existing one or two softest modes, in line with scenario (ii).

Notably, this type of intrinsic ability of the Hsp70 ATPase domain to undergo changes in its structure is consistent with the experimental observations made by Zuiderweg and co-workers [52]. Zuiderweg and co-workers determined by NMR residual dipolar coupling measurements the ensemble of structures sampled in solution by the ATPase domain of DnaK from *Thermus thermophilus* in the ADP-bound state. Interestingly, the conformational variabilities observed in this ensemble, as noted by the authors, were found to be consistent with the structural change crystallographically observed [18] in the ATPase domain upon NEF binding. This provides strong support, and experimental validation, for the intrinsic ability of the ATPase domain, in the absence of NEF, to have access to conformers that are pre-disposed to bind NEF, and for the utility of ANM analysis for accurately predicting the intrinsically favored changes in structure (softest modes).

### Sequence conservation

#### ET analysis highlights a cluster of conserved residues at the nucleotide-binding site

The results presented above lend strong support to the evolutionary selection/stabilization of a fold (by the Hsp70 ATPase domain) that endows suitable mobility and flexibilities at particular sites so as to favor functional changes in conformation (between open and closed forms), and optimal recognition and binding of the co-chaperones (NEFs). Next, we take a closer look at the evolutionary properties of Hsp70 ATPase domain *sequences*.

The results from ET analysis are presented in **Figure 3b**. Peaks therein represent the most conserved sites, within subfamilies (indicated by X), or across subfamilies (indicated by the single-letter amino acid code). See also *SM* **Figure S3** for an enlarged version of the same panel. The large majority, if not all, of the key residues reported in previous studies to be important to Hsp70 activity is captured by the ET peaks, including those participating in the hydrogen bond network proposed to form a proline switch (P147 and R155 in Hsc70; or their counterparts P143 and R151 in DnaK) [53]. Residues known to coordinate the nucleotides are shown in gray shade. As expected, most of these residues are highly conserved. Among them, G201 exhibits ET rank 1, succeeded by G338 and R342, and then G12. We also note among the peaks K71 and E175, two residues identified in our previous studies to play a key role in ATPase domain allosteric communication [45]. Residues involved in NEF recognition and binding, on the other hand, are colored red, orange, blue and green depending on their subdomains. These residues exhibit low levels of conservation.

#### A striking correlation between structural dynamics and sequence conservation

**Figure 5:**
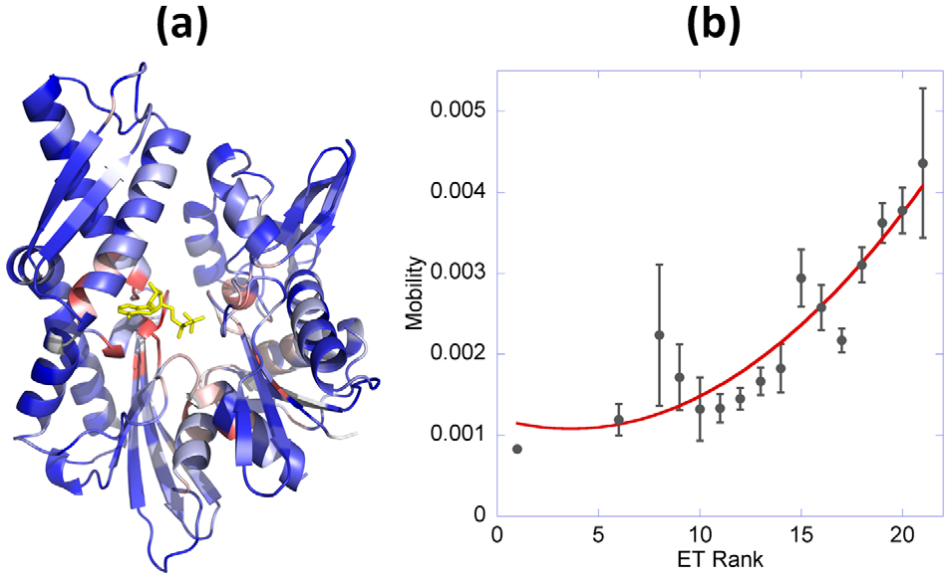
Correlation between residue mobility and its sequential variability. (**a**) Ribbon diagram colored by the ET rank of residues, from red (most conserved) to blue (most variable). (**b**) The average mobility of residues corresponding to different ET ranks. The mobilities are evaluated using Equation 1. The bars display the standard error for each ET rank. Best fitting second order polynomial (red curve) is shown to guide the eye (correlation coefficient of 0.92). See also *SM* **Figure S7** panel **b** for a similar plot, based on ConSurf score (instead of ET rank).

The color-coded ribbon diagram in **Figure 5** (based on the ET displayed in **Figure 3b**) shows that conserved residues (colored red) are mostly located in the nucleotide-binding pocket. The comparison of the weighted-average mobility profile in **Figure 2b** and the ET trace in **Figure 3b** suggests an inverse correlation between the extent of mobility of a given residue and its level of conservation: the ET trace indeed exhibits high peaks not only at nucleotide-binding sites, but also at other sites indicated by the GNM to participate in global hinge motion.

Towards a more detailed examination of this tendency, we have grouped residues based on their ET ranks, starting from the most conserved residues (ET rank = 1), and computed the average mobility profile of residues for each ET rank. **Figure 5b** displays the resulting relation between sequence conservation (ET rank) and global mobility. The ordinate represents the average displacement <*M*|_1–10_>_ET_ for all residues that exhibit a given ET rank (abscissa), and the bars display the standard error in each case. The observed decrease in mobility with increased conservation suggests that constraints on the collective mechanics of the molecule may be as important as those associated with chemical activity, such that the residues at key mechanical sites also tend to be evolutionarily conserved.

A closer examination (*SM* **Figure S7**) shows that G34, D292 and L274 are outliers when comparing their ET rank with their global mobility (they are too mobile for their level of conservation). Their enhanced mobilities may however be explained by their functionalities: G34 is presumably critical to maintaining the loop structure near the nucleotide-binding site; D292 is a class-specific residue recognized to be a key element of the signature loop that differentiates subfamilies [54]. It takes part in conserved salt-bridges with NEF basic residues in mammalian homologues (K238 for BAG; K245 for BP1).

L274 is located at the C-terminus of helix 9 near the nucleotide-binding site, and may be playing a key role in stabilizing this long helix in a functional state. This helix indeed appears to be bridging between the NEF-contacting residues on subdomain IIB and the nucleotide-binding residues in the central cleft, hence its high conservation (or high ET rank). E268 and R272 are two other residues on helix 9 in contact with both NEF and nucleotide, and as such, they may be playing a role in initiating the allosteric communication between the bound NEF and the nucleotide-binding pocket. Like most of other nucleotide-binding residues, E268 is relatively conserved (ET rank = 11) (**Figure 3b**); R272, on the other hand, is highly variable (ET rank of 17) and more exposed. Its high correlation with other NEF-contacting residues (discussed in the next section) and the orientation of its side chain (exposed) support its primary role in NEF recognition rather than nucleotide interaction.

As an additional verification of the relationship detected between collective mechanics and evolutionary conservation, we have examined the mobilities of residues as a function of their ConSurf scores. ConSurf scores provide a measure of the level of conservation, higher scores corresponding to less conserved residues (similar to ET rank) [32]. The plot in *SM* **Figure S7** also confirms the relation between the extent of restrictions in mobility and the level of conservation, again suggesting that sequential and structural variabilities go hand in hand.

### Sequence correlations

#### MI analysis highlights co-evolutionary patterns for NEF-recognition residues

**Figure 6:**
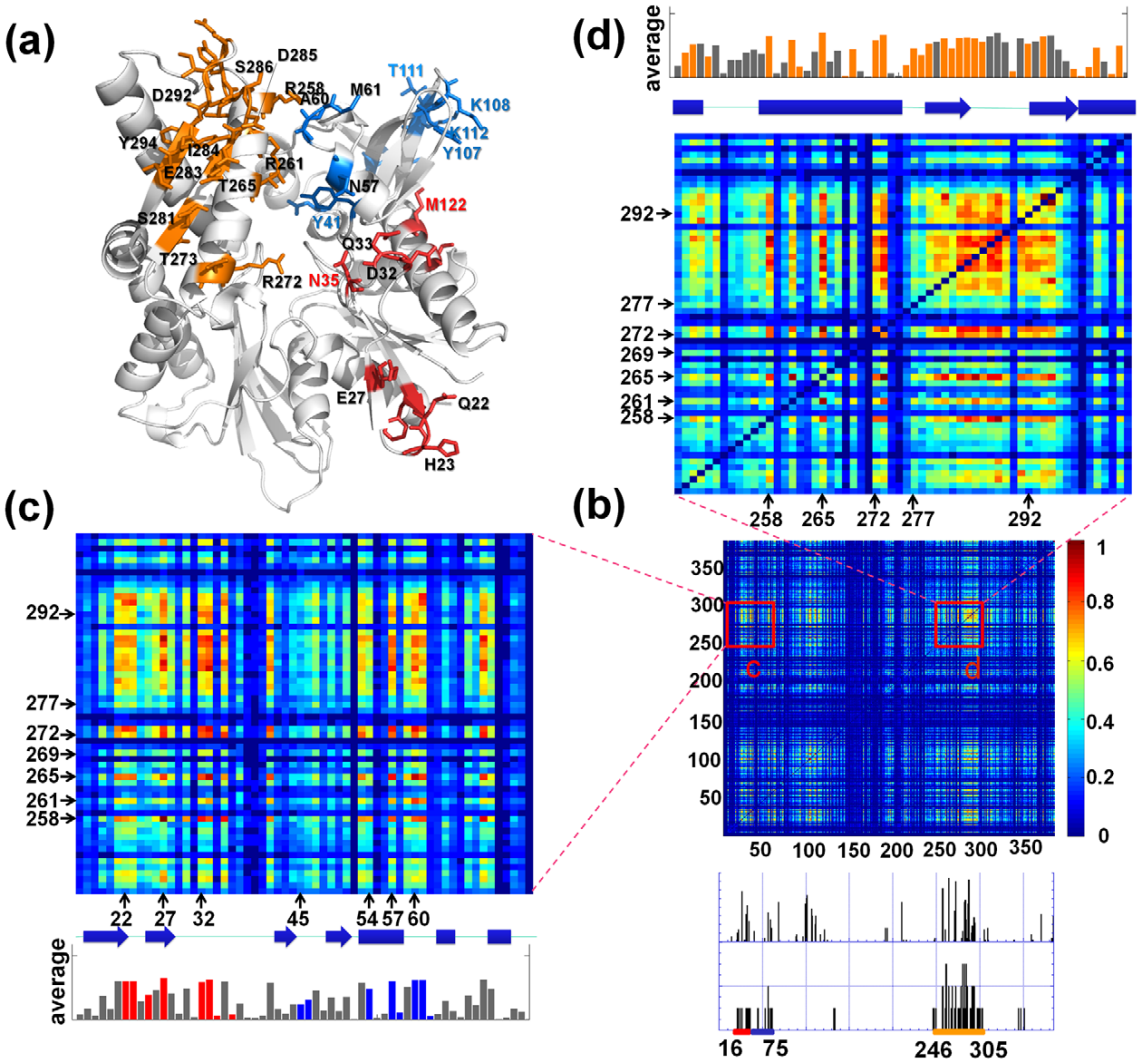
Co-evolution of NEF-binding residues revealed by mutual information (MI) maps constructed for the Hsp70 ATPase domain. (**a**) Amino acids distinguished by their high co-evolutionary patterns in the maps **c** and **d** (residues with average MI value greater than 0.32), shown in stick representation and colored by subdomains. Among them, the NEF contacting residues (Table S1) are labeled black, and others are colored by subdomain. Note the large proportion of charged or polar residues. (**b**) MI map for the ATPase domain sequence included in the MSA (residues 6–385). The color-coded bar on the right indicates the level of correlation between the evolution of residue pairs. Two regions containing a large number of NEF-binding residues are enlarged in panels **c** and **d**. The bar plots under the map display the contribution of each residue to the most correlated residue pairs (top 1%, 720 pairs) in the MI matrix (*upper plot*), and the frequency of NEF-ATPase domain contacts in three mammalian complexes (*lower plot*). (**c**) and (**d**) Close-up views of the MI map portions between residues 246–305 (containing the helix 9 and β-sheet E of subdomain IIB) and residues 16–75 (containing NEF-contacting segments in subdomains IA and IB) (**c**), and within residues 246–305 (**d**). The corresponding secondary structural elements are indicated along the abscissa by cylinders (α-helices) and arrows (β-strands). The bar plots display the average MI per residue, NEF binding residues being colored by their subdomain.

**Figure 7:**
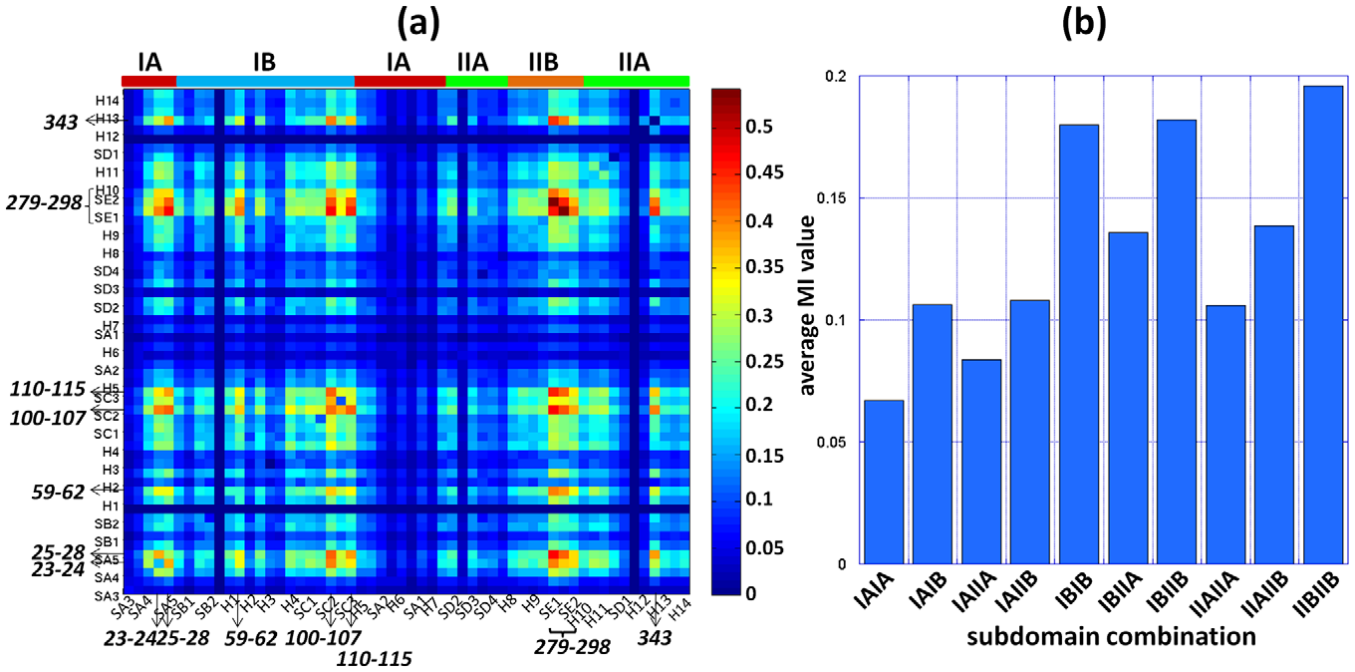
Average MI values calculated for different structural elements (helices/strands) and for different subdomains. The panels demonstrate the mean MI value between and within (**a**) pairs of secondary structure elements (the names of helices and sheets are based on the PDB entry 1HPM, H: α-helix, S: β-sheet) and (**b**) pairs of subdomains.

The results from the MI analysis of the 1627 Hsp70 ATPase domain sequences examined here are presented in **Figures 6** and **7**. The ribbon diagram in panel **a** highlights the residues distinguished by their co-evolutionary patterns. These are determined by analyzing the MI map for the complete sequence shown in **Figure 6b**. Close-up views of the two highlighted regions that contain the large majority of NEF-binding residues are presented in panels **c** and **d**. These two regions (residues 246–305 and 16–75) include 90% of all NEF-contacting residues. The bar plots below the MI map in panel **b** indicate the contribution of individual residues to the most correlated pairs in the MI matrix (*upper plot*), and the frequency of NEF-ATPase domain contacts made by these residues in the examined three mammalian complexes (*lower plot*). The bar plots and enlarged panels **c** and **d** clearly show that NEF-contacting residues exhibit high sequence correlations. Residue pairs that exhibit the highest MI values are listed in *SM* **Table S3**.

**Figure 7**, on the other hand, provides a broader view of co-evolution patterns for Hsp70 ATPase structural elements. Here average MI values are displayed for all pairs of secondary structural elements and loop regions (panel **a**), and all pairs of subdomains (panel **b**). The strong co-evolutionary property of residues within the subdomain IIB is clearly seen from panel **b**, succeeded by that within subdomain IB. Among the inter-subdomain correlations, we distinguish the pair of subdomains IB and IIB. Consistent with these patterns, four groups of secondary structural elements are distinguished in panel **a** by their most correlated evolutions: sheet E and connecting loop (Q279-T298) in subdomain IIB, the β-strand R100-Y107, and loop (V59-N62) between helices 1 and 2 in subdomain IB, and a β-strand and preceding turn (His23-Ile28) in subdomain IA.

**Figure 6d** describes in more detail the co-evolutionary properties within the subdomain IIB. The bar plots along the upper abscissa indicate the average MI values corresponding to each residue. The residues involved in NEF recognition are colored by the Hsp70 ATPase domain subdomain to which they belong. We note in particular the remarkably high MI values corresponding to the pairs of residues within the β-sheet E, except for the discontinuity at the loop residue G290. Furthermore, these residues display remarkably high co-evolutionary patterns with amino acids on helix 9 (K257-S275). Examples of such highly correlated pairs are R258-Y288, T265-D285 and T273-Y288 (ranking 5^th^, 6^th^, and 27^th^ respectively among all MI pairs, see **Table S3**). Notably, helix 9 also contains highly conserved residues (E268, K271 and S275) involved in nucleotide binding. This combination of co-evolving (NEF-recognition) and conserved (nucleotide-binding) residues endows helix 9 with a unique mediating role between the NEF-binding region and the nucleotide-binding pocket. Notably, the two β-strands and preceding α-helix on subdomain IIB emerge as a co-evolved structural entity, distinguished by its NEF-recognition and binding role, reminiscent of the functional ‘sectors’ pointed out by Ranganathan and coworkers [55] for S1A serine proteases.

**Figure 6c** reveals the cross-correlations between the evolutionary trends of subdomain IIB residues and the residues 16–75 on subdomains IA and IB. The above noted (β-hairpin and α-helix 9) residues of subdomain IIB appear to have co-evolved with well-defined residues on subdomains IA and IB. In particular the pairs E27-R258, E27-Y288, Q33-T273 and Q33-E283, exhibit remarkably high correlations (see **Table S3**), despite their long distance separation on the structure.

The observed sequence correlations may arise from several reasons [37], [55], [56] including those originating from the splits between subfamilies (phylogenetic noise). In particular, subdomain IIB has phylogenetic history contributing to its high variability, and subfamilies have evolved to partner with different NEFs. Regardless of the origin of these correlations, the MI map unambiguously shows that NEF-binding residues are distinguished by their co-evolutionary properties. As a further test, we performed a statistical coupling analysis [38] of sequence correlations (*SM* **Figure S8** and **Text S3**) which confirmed the results found from MI analysis, while the signals provided by MI are more pronounced due to the weighting strategy employed in SCA. Finally, we repeated the MI calculations (i) using an extended set of Hsp70 family sequences (retrieved from Pfam version 24), and (ii) allowing for larger fractions of gaps in the MSA. The results presented in the respective *SM* **Figure S9** and Figure **S10** corroborated the co-evolutionary patterns for subdomain IIB residues (see Also **Text S4**).

#### Complementary information provided by MI maps and ET analysis

The ET diagram (**Figure 3b**) and MI maps (**Figures 6** and **7**) provide complementary information. Those residues distinguished by their high conservation (peaks in **Figure 3b**) cannot usually be detected by the MI map, simply because they exhibit minimal, if any, mutations and it may be hard to capture their co-evolutionary couplings to other residues due to scarcity of data. For example, subdomain IA is known to be relatively more conserved as also confirmed by ET analysis (see the block of residues 140–185 belonging to this subdomain in **Figure 3b** or *SM* **Figure S3**), and the corresponding region in the MI map (**Figure 6b**) exhibit practically no signals indicative of correlated mutations. The less conserved subdomain IB, on the other hand, has several correlated residue pairs, including in particular those involved in NEF binding, which are furthermore correlated with the NEF binding residues on subdomain IIB.

Therefore, the sets of residues highlighted by these two analyses tend to be mutually exclusive, and involved in different roles, intrinsic (to ATPase domain, *per se*) *vs.* specific (to its interaction with different ligands/substrates such as NEFs). The structural regions where these two groups of residues are clustered and/or closely coupled (e.g., α-helix 9; see above) are suggested here to play a key role in reconciling the specific functions (e.g., NEF binding) of the Hsc70 ATPase domain with its intrinsic conserved properties (of nucleotide binding and ATP hydrolysis).

Yet, we note that in some cases some relatively conserved residues are also captured by their MI maps, because their (relatively infrequent but possible) mutations indeed require compensating mutations that can be detected, even if such mutations are rare. NEF binding R262 and D292 (with respective ET rank of 12 and 9) belong to this group of residues, and can sustain mutations provided that these are accompanied by compensating substitutions. As mentioned above R292 is a class-specific residue involved in salt-bridge formation with NEF residues, and likewise, R262 takes part in conserved interactions with acidic residues on NEF in mammalian homologues (D222 for BAG; E132 for BP1). Note that its counterpart in DnaK (R261) makes a contact with M174. This can be explained by the fact that binding of GrpE to DnaK is based on hydrophobic interactions instead of salt bridges [18].

## Discussion

### Mechanism of action of a modular domain: Interplay between structure-encoded global dynamics and sequence-specific local interactions

Organisms comply with the evolutionary pressure to maintain their phenotype by genotypic variations that are compensated or correlated as needed, conserving certain sequence fragments vital to preserving their functions [57]. Understanding the co-evolving and conserved sequence patterns in modular domains is an interesting problem in its own right [58], [59]. Understanding these patterns in the light of structural data, if available, provides us with further insights into shared mechanisms of interactions that form the molecular basis of the biological function of such modular domains. The Hsp70 ATPase domain is such a modular protein common to functionally diverse actin, hexokinase, and Hsp70 protein families [60]. The present combined analysis of structure-encoded dynamics and sequence evolution for Hsp70 ATPase domain discloses a subtle interplay between conserved interactions and those involving co-evolved residues. Conserved interactions define generic properties of the Hsp70 ATPase domain: these include the concerted dynamics of its four subdomains, which allow for sampling functional conformations (*e.g.*, that stabilized upon NEF binding, allowing for ADP release; shown in **Figure 3**), and the physicochemical events (ATP hydrolysis) at the nucleotide-binding site. Those residues involved in NEF recognition, on the other hand, show low-to-moderate conservation, but exhibit a remarkably high tendency to co-evolve, or undergo correlated mutations, again to achieve specific NEF-dependent recognition and binding activities. Interestingly, NEF residues that interact with the Hsp70 ATPase domain appear to be rather conserved (**Figure S11**) to maintain this specificity.

An observation of interest is the similarity between the interactions of the Hsp70 ATPase domain with different NEFs, in terms of structural dynamics. While Hsp70 ATPase domains are highly conserved both sequentially and structurally, the four NEFs examined have distinct structures and consequently different dynamics. The key point is that their binding to the ATPase domain involves in all cases the subdomain IIB of the ATPase domain, although not in exactly the same arrangement. Their binding to a common interfacial region on the ATPase domain point to a shared mechanism of interaction: The ATPase subdomain IIB is originally distinguished by its high mobility in the slowest mode, especially at the β-sheet E and the exposed loop connecting the two strands of this sheet; and after NEF binding, there is a significant suppression in its mobility. The conserved dynamics of the complexes suggests a role of subdomain IIB as an “adjustable handle”, which regulates the Hsp70 chaperone machine, to facilitate other proteins making use of its SBD.

Many applications using the ANM have shown that the substrate recognition involves a region distinguished by its enhanced mobility in the most cooperative (or softest) modes, which enables the molecule to optimize its interactions with the substrate. Here we can see that the C-terminal part of helix 8 and the loop of β-hairpin E enjoy this type of high mobility/adaptability. On the other hand, substrate ‘binding’ may also involve more constrained residues in the close neighborhood, which may play a role in transmitting allosteric effects. In the opposite case of a binding site composed exclusively of floppy residues, the structural changes induced upon substrate binding could dissipate locally and not efficiently transmitted. In this respect, we propose that the involvement of residues such as Arg258, Arg261 and Arg262 in subdomain IIB, or N57, A60 and M61 in subdomains IB is critically important in establishing the communication between subdomains and transmitting allosteric signals between NEF-binding and nucleotide binding sites.

A putative communication pathway that couples distant residues in different subdomains of the Hsp70 ATPase domain is suggested here by the structural mapping of correlated and conserved residues, which needs to be further established. **Figure 6a** displays those residues identified to be co-evolving. Notably, we observe several pairs making interdomain contacts, in addition to spatially distant residue pairs (*e.g.* H23 in subdomain IA and N57, A60 and M61 in subdomain IB correlated with R258, R261, E283 and D292 in subdomain IIB). In a recent study, R272, R261, Y15 and Y41 have been identified to play a central role in establishing the allosteric communication in the unbound Hsp70 ATPase domain, along with highly conserved residues K71, R72, E175 and H227 [45]. It remains to be seen if these central residues play a key role in mediating between these co-evolving, spatially distant residues. We also note that Smock et al. recently identified a sparse but structurally contiguous group of co-evolving residues at the interface between the ATPase domain and the SBD in Hsp70/110 protein family, which has been proposed to underlie the inter-domain allosteric coupling [61], in support of the role of co-evolved residues in mediating allosteric signaling.

### Pre-existing paths of reconfiguration intrinsic to Hsp70 ATPase domain fold accommodate binding of co-chaperones

Many recent studies have pointed out the validity of “pre-existing equilibrium” concept where a substrate or ligand simply selects from amongst an ensemble of conformations already accessible to the protein prior to binding [49], [62]–[67]. The present results, and recent applications of ENMs, suggest that more important than the pre-existence of these ‘states’, is the existence of energetically accessible ‘paths’ that provide access to those states, or the intrinsic tendency of the native structure to reconfigure towards such functional states. In terms of energy landscape description, what is needed is not the existence of multiple minima, the depths of which change upon ligand or substrate binding, but the existence of one or more directions of reconfigurations, or paths along the energy landscape, that are easily accessible to the protein and lead to the targeted (functional) conformer. The softest modes provide such paths. They define directions of motion in the space of collective coordinates, which incur a minimal energy ascent as the molecule moves away from its original energy minimum. They also present the best mechanisms of dissipating energy, if the system is perturbed. These are the modes that are being exploited when proteins bind ligands or substrates. Notably these functional conformations accessible near the native state can be observed by NMR residual dipolar coupling, as shown for Hsp70 ATPase domain by Zuiderweg and coworkers [52]. **Figure 4** clearly shows that movements along a handful of modes satisfactorily ensures the passage to the alternative (functional) open form, and that the open form itself has a strong tendency to restore its conformation back to the closed form, in the absence of NEF.

### Bridging between residue conservation and global dynamics

Protein-ligand binding interfaces and protein-protein contact interfaces are characterized by different sequence variation patterns. The protein-protein contact interfaces usually expose larger contact areas [68] and exhibit high mutation rates. Moreover, if the contact interface is a common recognition site for multiple targets (possibly in different organisms), co-evolution is likely to occur among the binding residues to preserve specific interactions and conformations at the sequence motif. On the other hand, the protein-ligand interface is usually buried in the folded core of the protein; in contrast to protein-protein interaction, the protein-ligand interaction is usually characterized by higher specificity, requiring sequence conservation [28], [29].

The Hsp70 ATPase domain exhibits patterns in close agreement with these general features: Its ligand (nucleotide) binding site essentially consists of highly conserved residues, which not only precisely coordinate the ligand, but also take part in a global hinge-bending region so that they are both chemically and mechanically required to be highly conserved. NEF recognition sites, on the other hand, exhibit much lower conservation properties; and in addition to their sequence variability, the subdomain IIB, which is observed to be most often involved in NEF binding, enjoys enhanced mobility. Briefly, global dynamics requirements entail residue conservation, and specific recognition entails sequence variation along with enhanced mobility. However, neither the sequence variability, nor the conformational mobility at NEF recognition sites, is random. The sequence variability takes place under unique restrictions, compensating mutations, as unraveled by the MI map. Conformational variability, on the other hand, is uniquely defined by the ATPase architecture, and precisely adept to accommodate the passage to the functional open state that is stabilized upon NEF binding. The ATPase domain uniquely juxtaposes such structure-encoded dynamics and sequence-specific interactions, which underlie its ubiquitous activities.

In general, subdomains IA and IIA are more conserved and more rigid than subdomains IB and IIB [69], as also indicated by the ET in **Figure 3b**; notably, they also serve as binding site to a number of proteins. For example, subdomain IA accounts for the binding of J-domain proteins [70]; subdomain IIA is reported to contain a putative binding site near its interface with subdomain IA (V189-V195) to the chaperonin-containing TCP-1 [71], and it is connected to the SBD by an inter-domain linker, which is considered important for the allosteric interactions between the two domains [72], [73]. It remains to be seen if the correlated sites on Hsp70 ATPase domain emerging from the MI analysis play a role in the functional communication with other co-chaperones or the SBD. Extensive experimental studies have been performed to date with the *E. coli* Hsp70, DnaK, to understand the molecular mechanism of activity of the molecular chaperones in the Hsp70 family. The analysis in the present paper will guide our interpretation of the NMR, FRET, and EPR data on different states accessible to DnaK. Each of these methods gives us a different window into the ensemble of conformational states populated in response to ATP, ADP and NEFs. Excitingly, a detailed chemical shift analysis of six different ligand bound states for the nucleotide-binding domain of DnaK, with and without the linker that connects it to the substrate-binding domain (i.e., 12 NMR samples compared pairwise and as a group) has pointed to the same subdomain interface rearrangements indicated in the present study (Zhuravleva & Gierasch, in preparation). Moreover, the NMR results point to the fundamental feature that subdomain IIB can undergo a hinge-like movement to enable nucleotide entry and release. It is this fundamental movement, intrinsic to Hsp70 ATPase domains, that different NEFs have exploited. They bind in different, sequence-specific ways, but modulate the same fundamental movement. Further detailed analysis of the ensemble distributions and rates of interconversion between states can be achieved using a synergistic battery of computational and experimental tools.

## Supporting Information

Table S1 ATPase domain residues making close atom-atom contacts with different NEFs. (a) Close atom-atom contacts are defined as those having interatomic distance less than 4 Å. (b)Amino acids are grouped according to their subdomain locations; those written in boldface are also detected by SASA calculations (Table S2) to exhibit a decrease in their accessible surface upon NEF binding. (c) The entries in parentheses refer to the aligned residues in the mammalian Hsp70s. (d) The original structure of Hsc70+HspBP1 complex only contains lobe II. (e) This complex contains four additional interfacial residues, all in subdomain IIA: Lys345, Lys348 and Asp352.
(0.06 MB DOC)

Table S2 Hsp70 ATPase domain residues exhibiting change in the solvent-accessible surface area (SASA) upon binding NEF. We calculated the solvent accessibility surface area (SASA) of each residue of the Hsp70 NBD for both the NEF-bound and -unbound forms, denoted by SASAapo and SASAbound, respectively. The change induced upon NEF-binding is designated as ΔSASA = SASA_bound_−SASA_apo_. Calculations were performed using PyMol *get_area* function. All residues with ΔSASA<0 are listed below. The unit for the surface area is Å^2^. We also listed in the 5th column the identity of NEF residues that make contacts (interatomic distance of <4 Å) with the ATPase domain residue listed in the 1st column. Note that all these residues are a subset of the residues listed in Table S1. Residue pairs that form salt bridges at the interface are written in italic. We note the abundance of such interactions in the mammalian chaperones/co-chaperone interfaces.
(0.09 MB DOC)

Table S3 Residue pairs distinguished by their sequence correlation (MI values above 0.8). Residue pairs are separated by at least two amino acids along the sequence.
(0.06 MB DOC)

Text S1 Smith-Waterman threshold score and percent identity of aligned sequences.
(0.05 MB DOC)

Text S2 Reconstructing the missing lobe I in the ATPase+NEF structure.
(0.05 MB DOC)

Text S3 Comparison of MI values with results from statistical coupling analysis.
(0.05 MB DOC)

Text S4 Evaluation of MSA quality.
(0.04 MB DOC)

Figure S1 Smith-Waterman threshold score and percent identity of aligned sequences. *Left*: Correlation between the SW score and the percent sequence identity of aligned sequences based on four different definitions, shown in panels (a)–(d); (e): distribution of sequence identity with respect to the reference sequence Hsc70, evaluated for the 4839 sequences retrieved from the Pfam v22 database for the Hsp70 family. The subset of sequences included in our MSA is shown by the upper bracket.
(0.07 MB PNG)

Figure S2 Reconstruction of the ATPase domain complexed with HspBP1. 1XQS is colored cyan, and 1S3X is colored orange.
(0.55 MB PNG)

Figure S3 High resolution version of panel (b) in the main text Figure 3.
(0.35 MB PNG)

Figure S4 Intrinsic mobilities of residues in the ATPase domain. (a) The profiles represent the GNM-predicted weighted average mobilities (squared) of all residues, as driven by the first ten slowest modes, calculated for the three structures of mammalian homologs of Hsp70 listed in the inset (see also rows 2–4 in Table S1). The profiles are normalized such that the area under each curve is 1. The thick black curve corresponds to the unbound form. (b) GNM-predicted weighted average mobilities of all residues, as driven by the first ten slowest modes, calculated for the structure of DnaK bound with GrpE.
(0.15 MB PNG)

Figure S5 Global dynamics of the ATPase domain. (a) Ribbon diagram of the ATPase domain in the unbound state color-coded by the mobilities in the first (lowest frequency, largest amplitude) GNM mode. Figure generated with PDB entry 1HPM. The slowest mode of the ATPase domain complexed with NEF is displayed for four different cases: (b) DnaK in contact with GrpE. (c) Hsc70 in contact with BAG-1. (d) Hsc70 in contact with Sse1. (e) Hsc70 in contact with HspBP1. Structural diagrams are generated with PDB entries (b) 1DKG (c) 1HX1 (d) 3D2E (e) 1XQS. The ATPase domain backbones are shown in stick representation, all in the same orientation, and the NEFs, as ribbon diagrams. In each case the complex is color-coded according to mobility (see the scale at the bottom).
(0.82 MB PNG)

Figure S6 Comparison of experimentally observed and computationally predicted structural changes in ATPase domain. The experimentally observed changes refer to the structural difference between the sse1-bound and -free forms of the NBD (respective PDB files 3D2E and 1HPM). The computational results are obtained by the ANM applied to the respective two structures. (a) Structural alignment of NEF-bound and unbound ATPase fragments. The unbound ATPase fragment (1HPM) is colored gray. The NEF-bound ATPase fragment (3D2E is color-coded according to its extent of deformation with respect to the unbound ATPase, the regions showing the largest deformation being colored red, and those unchanged, blue. The distance between Cα atoms of Ala60 and Arg258 is 5.0 Å in the closed form and 19.0 Å in the open form. Panels (b) s displays the results for the unbound (black curves) and Sse1-bound (red curves) ATPase domain. The solid thick curves with the squares represent the correlation cosine between the deformation vector d and the ANM modes (eigenvectors). The thin curves with the circles describe the cumulative overlap (Eq (2)). The results show that a subset of 9 slow modes accessible to the unbound form (panel b) ensures the passage to the NEF-bound conformer with an overlap of 0.82. The NEF-bound form exhibits an even stronger potential to be reconfigured back into its closed form, consistent with the preferred conformation of the NBD in the unbound form: top ranking mode yield an overlap of 0.92 with the experimental deformation vector *d*.
(0.17 MB PNG)

Figure S7 Comparison of residue mobilities with their evolutionary conservation properties. (a) The mobility for each residue averaged ver the first 10 GNM modes is plotted against its ET rank. Three outliers for ET rank 6, 8 and 9 are labeled, two of which (Gly34 and Asp292) are NEF-contacting residues. (b) Proportionality between the discretized ConSurf score and the average mobility (average <*M*>|_1–10_) for residues with the same discrete ConSurf score. The discretization is performed by sorting all residues according to the ConSurf score, grouping every 20 consecutive residues and evaluating the mean mobility for each group. The correlation coefficient between average mobility and discrete ConSurf score is 0.88.
(0.14 MB PNG)

Figure S8 Comparison of correlated mutations map obtained by MI analysis and by the SCA. The first two panels are the correlation maps calculated using (a) SCA and (b) MI. (c) The SCA correlation map after hierarchical clustering (note that the abscissa does not correspond to sequential residues anymore, but those rank-ordered according to their extent of correlated mutations. (d) The MI correlation map with residues re-ordered according to the same permutation in panel (c).
(2.18 MB PNG)

Figure S9 Comparison of MI matrices. Comparison of MI matrices obtained with (a) the original dataset of Hsp70 family sequences (4839 of them), retrieved from Pfam release 22, and (b) the larger dataset retrieved from the Pfam release 24.
(1.08 MB PNG)

Figure S10 MI Results from MSA with different gap tolerances. The upper panels, (a) (b) and (c), display the MI matrices calculated by allowing sequences with different coverages of the reference sequence, i.e. 2%, 25% and 50% gaps. Panels (d) (e) and (f), show the number of gaps observed in each column of the three respective MSAs.
(1.69 MB PNG)

Figure S11 ConSurf score for residues in each NEF. Lower score corresponds to higher conservation. Residues that are in contact with the Hsp70 ATPase domain are colored green.
(0.14 MB PNG)
