# Supplementary material for: Role of Hsp70 ATPase Domain Intrinsic Dynamics and Sequence Evolution in Enabling its Functional Interactions with NEFs
Source: PLoS Comput Biol. 2010 Sep 16;6(9):e1000931. doi: 10.1371/journal.pcbi.1000931 (PMC2940730; doi:10.1371/journal.pcbi.1000931)
Supplement: Table S1 — ATPase domain residues making close atom-atom contacts with different NEFs. (a) Close atom-atom contacts are defined as those having interatomic distance less than 4 Å. (b)Amino acids are grouped according to their subdomain locations; those written in boldface are also detected by SASA calculations (Table S2) to exhibit a decrease in their accessible surface upon NEF binding. (c) The entries in parentheses refer to the aligned residues in the mammalian Hsp70s. (d) The original structure of Hsc70+HspBP1 complex only contains lobe II. (e) This complex contains four additional interfacial residues, all in subdomain IIA: Lys345, Lys348 and Asp352. (0.06 MB DOC) [file pcbi.1000931.s001.doc]

# Table S1. ATPase domain residues making close atom-atom contacts with different NEFs ^(a)^

| PDB ID  [ref] | Molecule and  Organism | | NEF | NEF-Recognition/Binding Residues^(b)^ | | |
| --- | --- | --- | --- | --- | --- | --- |
|  |  |  |  | Subdomain IIB | Subdomain IA | Subdomain IB |
| 1DKG[1] | DnaK | *E.coli* | GrpE | **L257 (R258)**, **Q260 (R261)**, **R261 (R262)**, **E264 (T265)**, N282 (E283), P284 (D285), Y285 (S286) ^(c)^ | E28 (A30), E31 (Q33), E128 (E132), E129 (A133), **Y130 (Y134)**, L131 (L135), **G132 (G136)** ^(c)^ | L49 (L50), **P53 (A54)**, **R56 (N57)**, Q57 (Q58), **V59 (A60), T60 (M61)** ^(c)^ |
| 1HX1  [2] | Hsc70 | Bovine | BAG-1 | **R258**, **R261**, **R262**, **T265**, R269, S281, **E283**, I284, D285, **S286**, G290, **D292**, Y294 |  | F45, **D46**, **N57**, A60-N62 |
| 1XQS  [3] ^(d)^ | Hsc70 | Human | HspBP1 | **R247**, **K248**, K250, **R258**, **R262**, T265, **E268**, **R269**, **R272**, **T273**, **S277**, **Q279**, **S281**, R282, **E283**, **D285**, **D292**, **Y294** |  |  |
| 3D2E  [4]^(e)^ | Hsc70 | Human | Sse1 (Hsp110) | R262, T273, S276, T278, Q279, S281, E283, **D285**, S286, **T298**, R299, **A300**, R301, Glu303, **E304** | Q22, **H23**, K25, E27, D32-G34, R36, A133-**Y134** | **A54**, **N57**, Q58 |

1. Close atom-atom contacts are defined as those having interatomic distance less than 4 Å.
2. Amino acids are grouped according to their subdomain locations; those written in boldface are also detected by SASA calculations (**Table S2**) to exhibit a decrease in their accessible surface upon NEF binding.
3. The entries in parentheses refer to the aligned residues in the mammalian Hsp70s
4. The original structure of Hsc70+HspBP1 complex only contains lobe II.
5. This complex contains four additional interfacial residues, all in subdomain IIA: Lys345, **Lys348** and Asp352.
