# Supplementary material for: Role of Hsp70 ATPase Domain Intrinsic Dynamics and Sequence Evolution in Enabling its Functional Interactions with NEFs
Source: PLoS Comput Biol. 2010 Sep 16;6(9):e1000931. doi: 10.1371/journal.pcbi.1000931 (PMC2940730; doi:10.1371/journal.pcbi.1000931)
Supplement: Table S2 — Hsp70 ATPase domain residues exhibiting change in the solvent-accessible surface area (SASA) upon binding NEF. We calculated the solvent accessibility surface area (SASA) of each residue of the Hsp70 NBD for both the NEF-bound and -unbound forms, denoted by SASAapo and SASAbound, respectively. The change induced upon NEF-binding is designated as ΔSASA = SASAbound−SASAapo. Calculations were performed using PyMol get_area function. All residues with ΔSASA<0 are listed below. The unit for the surface area is Å2. We also listed in the 5th column the identity of NEF residues that make contacts (interatomic distance of <4 Å) with the ATPase domain residue listed in the 1st column. Note that all these residues are a subset of the residues listed in Table S1. Residue pairs that form salt bridges at the interface are written in italic. We note the abundance of such interactions in the mammalian chaperones/co-chaperone interfaces. (0.09 MB DOC) [file pcbi.1000931.s002.doc]

# Table S2. Hsp70 ATPase domain residues exhibiting change in the solvent-accessible surface area (SASA) upon binding NEF

We calculated the solvent accessibility surface area (SASA) of each residue of the Hsp70 NBD for both the NEF-bound and –unbound forms, denoted by SASA_apo_ and SASA_bound_, respectively. The change induced upon NEF-binding is designated as ΔSASA = SASA_bound_ - SASA_apo_. Calculations were performed using PyMol *get_area* function. All residues with ΔSASA< 0 are listed below. The unit for the surface area is Å^2^. We also listed in the 5^th^ column the identity of NEF residues that make contacts (interatomic distance of < 4 Å) with the ATPase domain residue listed in the 1^st^ column. Note that *all* these residues are a subset of the residues listed in **Table S1**. Residue pairs that form salt bridges at the interface are written in italic. We note the abundance of such interactions in the mammalian chaperones/co-chaperone interfaces.

**Table S2.1 SASA change of DnaK ATPase domain residues upon binding GrpE**

| ***Residue(*)*** | ***SASA_apo_*** | ***SASA_bound_*** | ***−ΔSASA*** (Å^2^) | ***NEF Residues in contact*** |
| --- | --- | --- | --- | --- |
| Pro53 (Ala54) | 82.03 | 81.83 | 0.19 | Pro151 |
| Arg56 (Asn57) | 146.49 | 142.95 | 3.54 | Pro151, Asn152, His154, Gln155, Ala156, Ala187 |
| Val59 (Ala60) | 90.33 | 90.09 | 0.24 | Ala156 |
| Thr60 (Met61) | 90.27 | 86.92 | 3.36 | Ala156, Val192 |
| Tyr130 (Tyr134) | 141.06 | 140.15 | 0.91 | Arg74 |
| Gly132 (Gly136) | 51.14 | 48.70 | 2.45 | Arg74 |
| Leu257 (Arg258) | 110.30 | 109.38 | 0.92 | Ile157, Ala158, Met174 |
| Gln260 (Arg261) | 112.61 | 109.39 | 3.21 | Ile157 |
| Arg261 (Arg262) | 142.77 | 139.55 | 3.21 | Met174 |
| Glu264 (Thr265) | 108.62 | 102.98 | 5.64 | Gln155, Ile157, Met189 |
| *(*) in parentheses are their counterparts in the mammalian Hsp70 ATPase domain* | | | | |

**Table S2.2 SASA change of Hsp70 ATPase domain residues upon binding BAG-1**

| ***Residue*** | ***SASA_apo_*** | ***SASA_bound_*** | ***−ΔSASA*** (Å^2^) | ***NEF Residues in contact*** |
| --- | --- | --- | --- | --- |
| *Asp46* | 98.62 | 98.08 | 0.55 | *Arg205* |
| Asn57 | 97.46 | 97.27 | 0.19 | Lys216 |
| *Arg258* | 144.87 | 142.61 | 2.25 | Ile211, Met215, *Asp252* |
| *Arg261* | 142.13 | 138.27 | 3.87 | *Glu212*, Met215, *Glu219* |
| *Arg262* | 143.09 | 141.55 | 1.54 | Leu218, *Asp222*, Gln245 |
| Thr265 | 89.33 | 87.38 | 1.95 | *Glu219* |
| *Glu283* | 116.50 | 113.45 | 3.05 | Asp222, Leu224, Ile225, Leu226, *Arg237* |
| Ser286 | 72.65 | 68.72 | 3.94 | Val241, Lys242, Gln245 |
| *Asp292* | 99.76 | 98.65 | 1.10 | *Lys238* |

**Table S2.3 SASA change of Hsp70 ATPase domain residues upon binding HspBP1**

| ***Residue*** | ***SASA_apo_*** | ***SASA_bound_*** | ***−ΔSASA***(Å^2^) | ***NEF Residues in contact*** |
| --- | --- | --- | --- | --- |
| *Arg247* | 146.92 | 146.51 | 0.41 | *Glu332, Glu333,* Gln335*, Glu336* |
| *Lys248* | 126.95 | 121.21 | 5.75 | *Glu290, Glu333, Glu336* |
| *Arg258* | 147.39 | 145.15 | 2.24 | *Asp129* |
| *Arg262* | 141.13 | 139.86 | 1.27 | *Glu132* |
| Glu268 | 114.79 | 110.52 | 4.27 | Met134 |
| *Arg269* | 145.14 | 139.68 | 5.45 | Cys131, *Glu132*, Met134, Ala137, Thr171, Cys172, Ser173, Gln174, Asn175, Val176 |
| Arg272 | 143.13 | 140.21 | 2.92 | Met134 |
| Thr273 | 88.87 | 87.04 | 1.83 | Asn175, Glu218 |
| Ser277 | 75.78 | 75.23 | 0.55 | Glu218 |
| Gln279 | 112.57 | 111.05 | 1.53 | Glu218, Val259 |
| Ser281 | 76.19 | 72.83 | 3.36 | Asn175, Arg217, Phe252, Asn256 |
| *Glu283* | 117.18 | 114.37 | 2.82 | Gln174, Phe210, Ser213, *Arg217*, *Lys249*, Phe252 |
| *Asp285* | 98.37 | 96.22 | 2.16 | Gln167, *Lys207*, *Lys249* |
| *Asp292* | 95.86 | 91.71 | 4.15 | *Lys245* |
| Tyr294 | 149.11 | 147.31 | 1.79 | Val248, Lys249, Phe252, His291 |

**Table S2.4 SASA change of Hsp70 ATPase domain residues upon binding Sse1**

| ***Residue*** | ***SASA_apo_*** | ***SASA_bound_*** | ***−ΔSASA***(Å^2^) | ***NEF Residues in contact*** |
| --- | --- | --- | --- | --- |
| His23 | 121.85 | 121.04 | 0.81 | Phe185, Thr187 |
| Ala54 | 67.74 | 66.83 | 0.91 | Ala280, Asn281 |
| Asn57 | 98.54 | 96.39 | 2.15 | Val277, Ala280, Asn281 |
| Tyr134 | 145.56 | 145.19 | 0.36 | Thr364, Thr365, Leu366, Asn367 |
| *Asp285* | 98.16 | 97.30 | 0.86 | *Lys262* |
| Thr298 | 93.55 | 91.39 | 2.16 | Asn572, Glu575 |
| Ala300 | 68.16 | 67.46 | 0.71 | Asn572, Glu575, Glu576, Tyr579 |
| Glu304 | 114.83 | 114.26 | 0.57 | Tyr579 |
| *Lys348* | 119.51 | 118.44 | 1.08 | *Glu576* |
