# Supplementary material for: Role of Hsp70 ATPase Domain Intrinsic Dynamics and Sequence Evolution in Enabling its Functional Interactions with NEFs
Source: PLoS Comput Biol. 2010 Sep 16;6(9):e1000931. doi: 10.1371/journal.pcbi.1000931 (PMC2940730; doi:10.1371/journal.pcbi.1000931)
Supplement: Table S3 — Residue pairs distinguished by their sequence correlation (MI values above 0.8). Residue pairs are separated by at least two amino acids along the sequence. (0.06 MB DOC) [file pcbi.1000931.s003.doc]

# Table S3. Residue pairs distinguished by their sequence correlation (MI values above 0.8). (*)

| ***Residue pair*** | ***MI value*** |
| --- | --- |
| Asp97---Lys102 | 1.0106 |
| Thr265---Thr273 | 0.90944 |
| Glu27---Arg258 | 0.88516 |
| Arg261---Thr265 | 0.87809 |
| Arg258---Tyr288 | 0.86932 |
| Thr265---Asp285 | 0.85692 |
| Lys102---Thr295 | 0.85576 |
| Thr265---Ser385 | 0.85391 |
| Pro101---Arg258 | 0.84257 |
| Glu27---Asp69 | 0.84196 |
| Glu27---Tyr288 | 0.83461 |
| Arg100---Lys102 | 0.83161 |
| Glu27---Pro101 | 0.83021 |
| Lys102---Lys108 | 0.82885 |
| Gln33---Thr265 | 0.82781 |
| Tyr107---Thr265 | 0.82534 |
| Asp69---Pro101 | 0.82013 |
| Ala60---Thr265 | 0.81996 |
| His23---Lys102 | 0.81836 |
| Lys102---Tyr107 | 0.81686 |
| Ser281---Thr295 | 0.81408 |
| Thr265---Glu283 | 0.81156 |
| Arg100---Tyr288 | 0.81134 |
| Arg100---Arg258 | 0.8111 |
| Gln33---Glu283 | 0.80765 |
| Thr265---Ser281 | 0.8064 |
| Thr273---Tyr288 | 0.80549 |
| Thr265---Tyr288 | 0.80536 |
| Lys102---Tyr294 | 0.80341 |
| Tyr107---Tyr288 | 0.80332 |
| Glu27---Arg100 | 0.80155 |
| Gln33---Thr273 | 0.80068 |
| Asp69---Arg258 | 0.80065 |

(*) residue pairs separated by at least two amino acids along the sequence
