# Supplementary material for: Role of Hsp70 ATPase Domain Intrinsic Dynamics and Sequence Evolution in Enabling its Functional Interactions with NEFs
Source: PLoS Comput Biol. 2010 Sep 16;6(9):e1000931. doi: 10.1371/journal.pcbi.1000931 (PMC2940730; doi:10.1371/journal.pcbi.1000931)
Supplement: Text S1 — Smith-Waterman threshold score and percent identity of aligned sequences. (0.05 MB DOC) [file pcbi.1000931.s004.doc]

The determination of the optimal threshold score for selecting MSA sequences is non-trivial. In general, sequence identity below 40% implies functional divergence[1,2]. On the other hand, evolutionary information from important homologs such as DnaK (the bacterial homolog of Hsp70) may be lost upon removal of all sequences with sequence identity below this threshold. We present here the correlation between the sequence percent identity (PID) and the Smith-Waterman alignment (SW) score for the examined MSA. PIDs are calculated based on four alternative definitions [3], where the ratio of the sequence identity (numerator) to four different quantities are evaluated. The denominators used in the four different definitions are

1. Length of the shorter sequence (L1).
2. Number of aligned positions, i.e., alignment length (includes gaps, if any) (L2).
3. Number of aligned residue pairs, i.e., identities and nonidentities (excludes gaps, if any) (L3).
4. Arithmetic mean sequence length (L4).

Panels A-D in the **Figure S1** refer to these four respective definitions, and illustrate how each SW score maps to a distribution of sequence identities.

A threshold of 300 for SW alignment score was adopted to ensure that close DnaK orthologs were kept in the MSA while eliminating the homologs below 40% sequence identity, approximately. This threshold eliminated ~ 2/3 of the original set, to yield a MSA of 1627 sequences with 380 columns.

Reference List

1. Lichtarge O, Sowa ME (2002) Evolutionary predictions of binding surfaces and interactions. Curr Opin Struct Biol 12: 21-27

2. Olmea O, Rost B, Valencia A (1999) Effective use of sequence correlation and conservation in fold recognition. J Mol Biol 293: 1221-1239

3. May AC (2004) Percent sequence identity; the need to be explicit. Structure 12: 737-738
