# Supplementary material for: Role of Hsp70 ATPase Domain Intrinsic Dynamics and Sequence Evolution in Enabling its Functional Interactions with NEFs
Source: PLoS Comput Biol. 2010 Sep 16;6(9):e1000931. doi: 10.1371/journal.pcbi.1000931 (PMC2940730; doi:10.1371/journal.pcbi.1000931)
Supplement: Text S2 — Reconstructing the missing lobe I in the ATPase+NEF structure. (0.05 MB DOC) [file pcbi.1000931.s005.doc]

We used 1S3X as a “template” to replace lobe I in the original 1XQS structure. 1S3X was selected because of its highest sequence homology with respect to 1XQS (189 identical amino acids out of 190 in lobe I).

The reconstruction procedure was based on the superposition of C_α_ atoms of the matching residues (i.e., lobe I) in 1S3X and 1XQS, since the reconstructed structure is solely intended for analysis with coarse-grained models. Optimal match between the C_α_ atoms was obtained using the C alpha Match webserver [1].

After 1S3X was optimally superimposed onto 1XQS, the original lobe I in 1XQS was replaced by the complete ATPase domain. An illustration of the procedure is illustrated in the **Figure S2** below. Note that a closed-form was substituted for an open form in this reconstruction, which gave rise to steric clashes between a few atom pairs at the interface between the subdomain IB and HspBP1. These could be alleviated by minor side chain reorientations, and would have minimal, if any, effect on the slow modes obtained with the GNM, because the GNM analysis is based on the backbone topology as a whole (with little effect from inaccuracies in atomic coordinates that fall below the resolution of the model) or the distribution of C^α^-C^α^ contacts within a cutoff distance of 7-10 Å, and the global modes are robust to minor changes in structural coordinates.

Reference List

1. Fischer D, Bachar O, Nussinov R, Wolfson H (1992) An efficient automated computer vision based technique for detection of three dimensional structural motifs in proteins. J Biomol Struct Dyn 9: 769-789
