# Supplementary material for: Role of Hsp70 ATPase Domain Intrinsic Dynamics and Sequence Evolution in Enabling its Functional Interactions with NEFs
Source: PLoS Comput Biol. 2010 Sep 16;6(9):e1000931. doi: 10.1371/journal.pcbi.1000931 (PMC2940730; doi:10.1371/journal.pcbi.1000931)
Supplement: Text S3 — Comparison of MI values with results from statistical coupling analysis. (0.05 MB DOC) [file pcbi.1000931.s006.doc]

There have been a number of methods proposed for sequence co-evolution analysis [1] apart from MI used in the current study; one of the most well-known is the statistical coupling analysis (SCA) [2,3]. As a benchmark of performance, we performed SCA calculations (SCA version 3.0) on the same MSA that were used for MI analysis, and compared the results (Figure S7).

The correlation matrices calculated in both analyses show similar patterns. Subdomain IIB exhibits the highest degree of correlation with a wide range of residues in both cases; however, we notice that the signals in the MI analysis are more distinctive, whereas the range of values in the SCA matrix is range (0-4.8 as opposed to 0-1 in the MI matrix). In the clustered maps, the high correlation in certain regions of the MI matrix has been suppressed in the SCA matrix, which may be attributed to the noise reduction step in SCA. Overall, in both matrices the majority of NEF-contact residues are identified as highly correlated with each other.

Reference List

1. Fodor AA, Aldrich RW (2004) Influence of conservation on calculations of amino acid covariance in multiple sequence alignments. Proteins-Structure Function and Bioinformatics 56: 211-221

2. Lockless SW, Ranganathan R (1999) Evolutionarily conserved pathways of energetic connectivity in protein families. Science 286: 295-299

3. Suel GM, Lockless SW, Wall MA, Ranganathan R (2003) Evolutionarily conserved networks of residues mediate allosteric communication in proteins. Nature Structural Biology 10: 59-69
