# Supplementary material for: Role of Hsp70 ATPase Domain Intrinsic Dynamics and Sequence Evolution in Enabling its Functional Interactions with NEFs
Source: PLoS Comput Biol. 2010 Sep 16;6(9):e1000931. doi: 10.1371/journal.pcbi.1000931 (PMC2940730; doi:10.1371/journal.pcbi.1000931)
Supplement: Text S4 — Evaluation of MSA quality. (0.04 MB DOC) [file pcbi.1000931.s007.doc]

For further benchmarking the results we have repeated our calculations (i) using the more updated Pfam version 24 which contains 9069 sequences in the PF00012 family (**Figure S9**), and (ii) increasing our gap tolerance to 25% and 50% (**Figure S10**). Below are the results obtained in the respective analyses, which practically confirms the results presented in **Figure 6a** and **S8a-b**. Note that panels (**a**) and (**b**) in **Figure S9** are hardly distinguishable in **Figure S9**, confirming that the original dataset provides a good representation of the complete ensemble of Hsp70 sequences. **Figure S10** shows that with increasing gap tolerance, certain regions of the ATPase domain tend to accumulate gaps, e.g., the N-terminus and the residues 70-115 in subdomain IB; and the corresponding regions in the MI matrices exhibit stronger signals. On the other hand, the signals in subdomain IIB consistently remain distinguishable in all three cases, corroborating our previous results concerning the co-evolutionary patterns in this subdomain, around residues 246-305 in particular.
