# Supplementary figures and images for: Role of Hsp70 ATPase Domain Intrinsic Dynamics and Sequence Evolution in Enabling its Functional Interactions with NEFs
Source: PLoS Comput Biol. 2010 Sep 16;6(9):e1000931. doi: 10.1371/journal.pcbi.1000931 (PMC2940730; doi:10.1371/journal.pcbi.1000931)

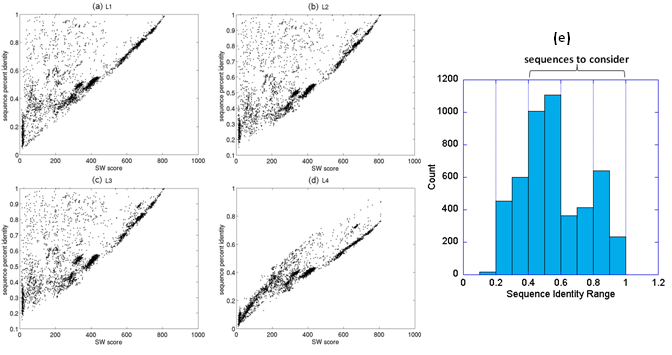

Supplement: Figure S1 — Smith-Waterman threshold score and percent identity of aligned sequences. Left: Correlation between the SW score and the percent sequence identity of aligned sequences based on four different definitions, shown in panels (a)–(d); (e): distribution of sequence identity with respect to the reference sequence Hsc70, evaluated for the 4839 sequences retrieved from the Pfam v22 database for the Hsp70 family. The subset of sequences included in our MSA is shown by the upper bracket. (0.07 MB PNG) [file pcbi.1000931.s008.png]

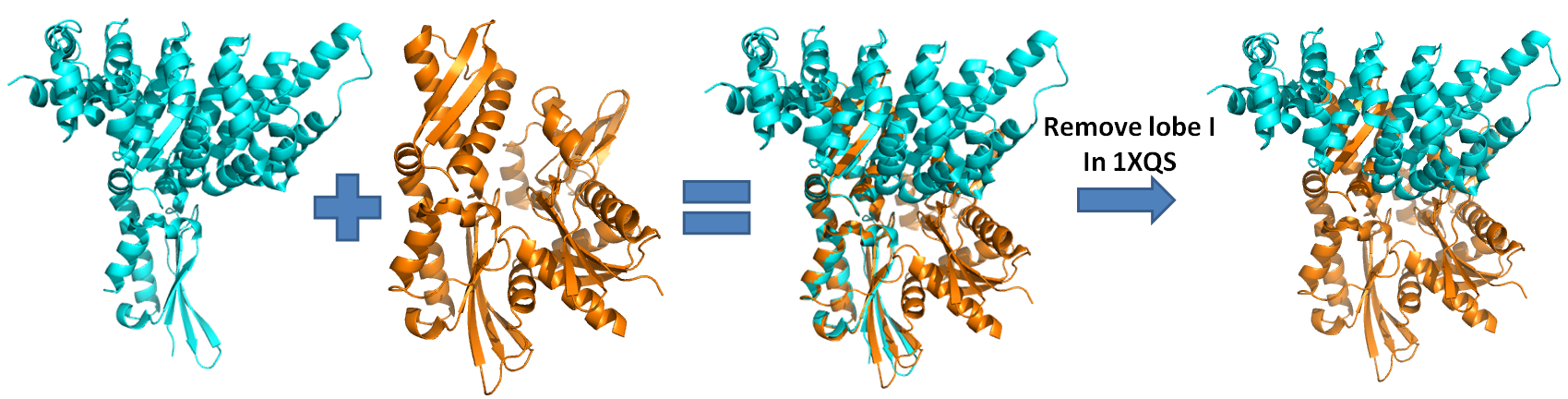

Supplement: Figure S2 — Reconstruction of the ATPase domain complexed with HspBP1. 1XQS is colored cyan, and 1S3X is colored orange. (0.55 MB PNG) [file pcbi.1000931.s009.png]

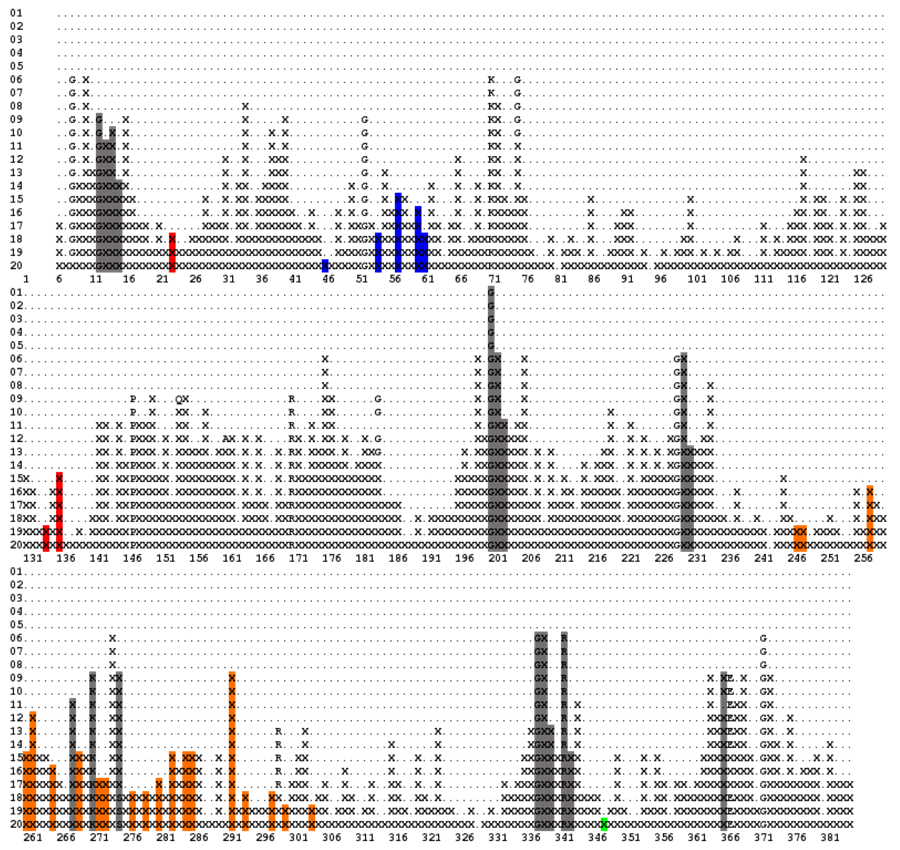

Supplement: Figure S3 — High resolution version of panel (b) in the main text Figure 3. (0.35 MB PNG) [file pcbi.1000931.s010.png]

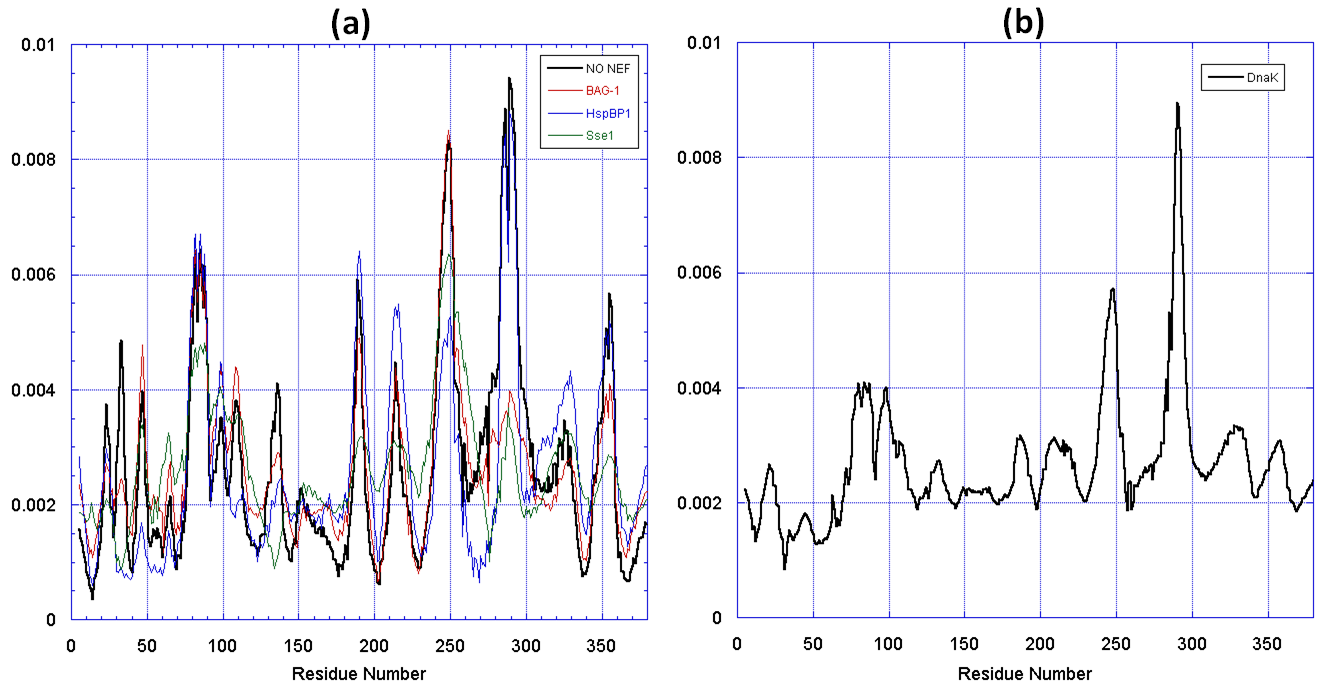

Supplement: Figure S4 — Intrinsic mobilities of residues in the ATPase domain. (a) The profiles represent the GNM-predicted weighted average mobilities (squared) of all residues, as driven by the first ten slowest modes, calculated for the three structures of mammalian homologs of Hsp70 listed in the inset (see also rows 2–4 in Table S1). The profiles are normalized such that the area under each curve is 1. The thick black curve corresponds to the unbound form. (b) GNM-predicted weighted average mobilities of all residues, as driven by the first ten slowest modes, calculated for the structure of DnaK bound with GrpE. (0.15 MB PNG) [file pcbi.1000931.s011.png]

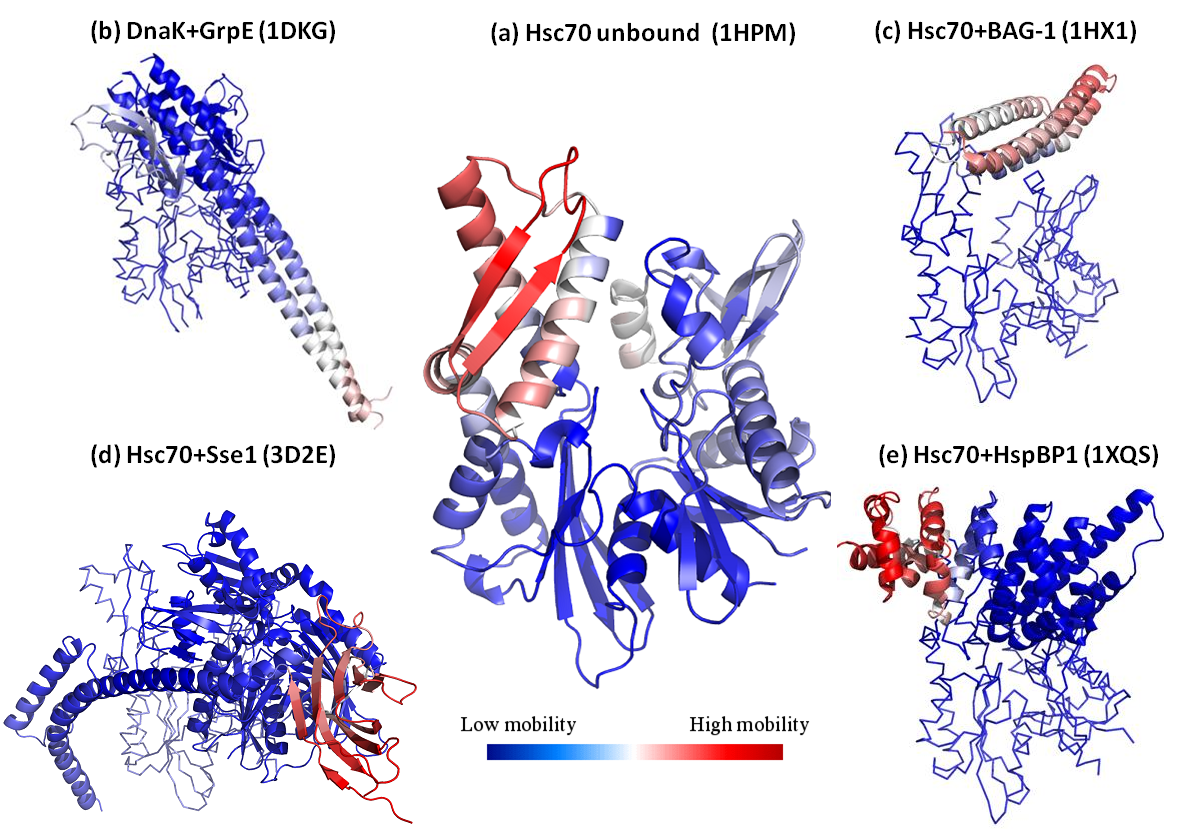

Supplement: Figure S5 — Global dynamics of the ATPase domain. (a) Ribbon diagram of the ATPase domain in the unbound state color-coded by the mobilities in the first (lowest frequency, largest amplitude) GNM mode. Figure generated with PDB entry 1HPM. The slowest mode of the ATPase domain complexed with NEF is displayed for four different cases: (b) DnaK in contact with GrpE. (c) Hsc70 in contact with BAG-1. (d) Hsc70 in contact with Sse1. (e) Hsc70 in contact with HspBP1. Structural diagrams are generated with PDB entries (b) 1DKG (c) 1HX1 (d) 3D2E (e) 1XQS. The ATPase domain backbones are shown in stick representation, all in the same orientation, and the NEFs, as ribbon diagrams. In each case the complex is color-coded according to mobility (see the scale at the bottom). (0.82 MB PNG) [file pcbi.1000931.s012.png]

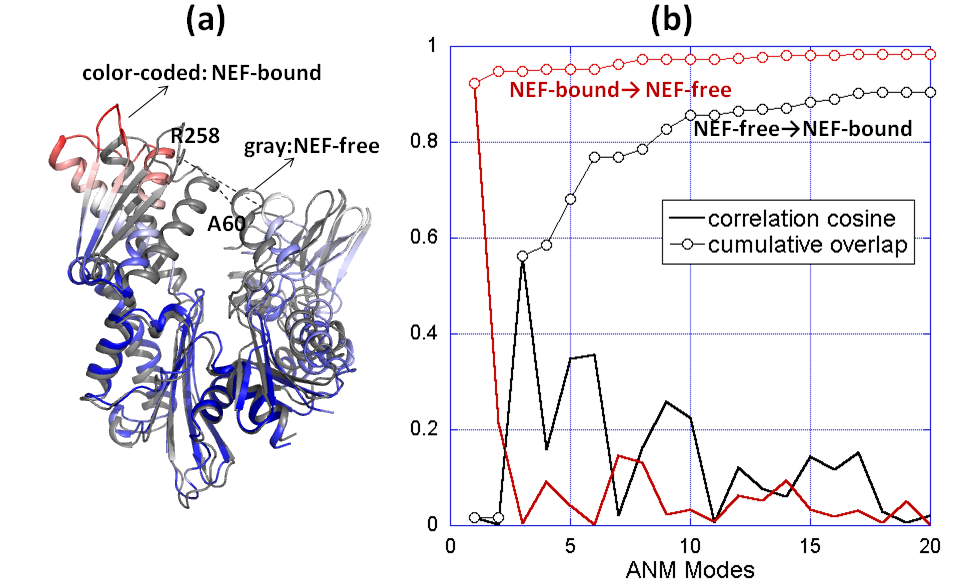

Supplement: Figure S6 — Comparison of experimentally observed and computationally predicted structural changes in ATPase domain. The experimentally observed changes refer to the structural difference between the sse1-bound and -free forms of the NBD (respective PDB files 3D2E and 1HPM). The computational results are obtained by the ANM applied to the respective two structures. (a) Structural alignment of NEF-bound and unbound ATPase fragments. The unbound ATPase fragment (1HPM) is colored gray. The NEF-bound ATPase fragment (3D2E is color-coded according to its extent of deformation with respect to the unbound ATPase, the regions showing the largest deformation being colored red, and those unchanged, blue. The distance between Cα atoms of Ala60 and Arg258 is 5.0 Å in the closed form and 19.0 Å in the open form. Panels (b) s displays the results for the unbound (black curves) and Sse1-bound (red curves) ATPase domain. The solid thick curves with the squares represent the correlation cosine between the deformation vector d and the ANM modes (eigenvectors). The thin curves with the circles describe the cumulative overlap (Eq (2)). The results show that a subset of 9 slow modes accessible to the unbound form (panel b) ensures the passage to the NEF-bound conformer with an overlap of 0.82. The NEF-bound form exhibits an even stronger potential to be reconfigured back into its closed form, consistent with the preferred conformation of the NBD in the unbound form: top ranking mode yield an overlap of 0.92 with the experimental deformation vector d. (0.17 MB PNG) [file pcbi.1000931.s013.png]

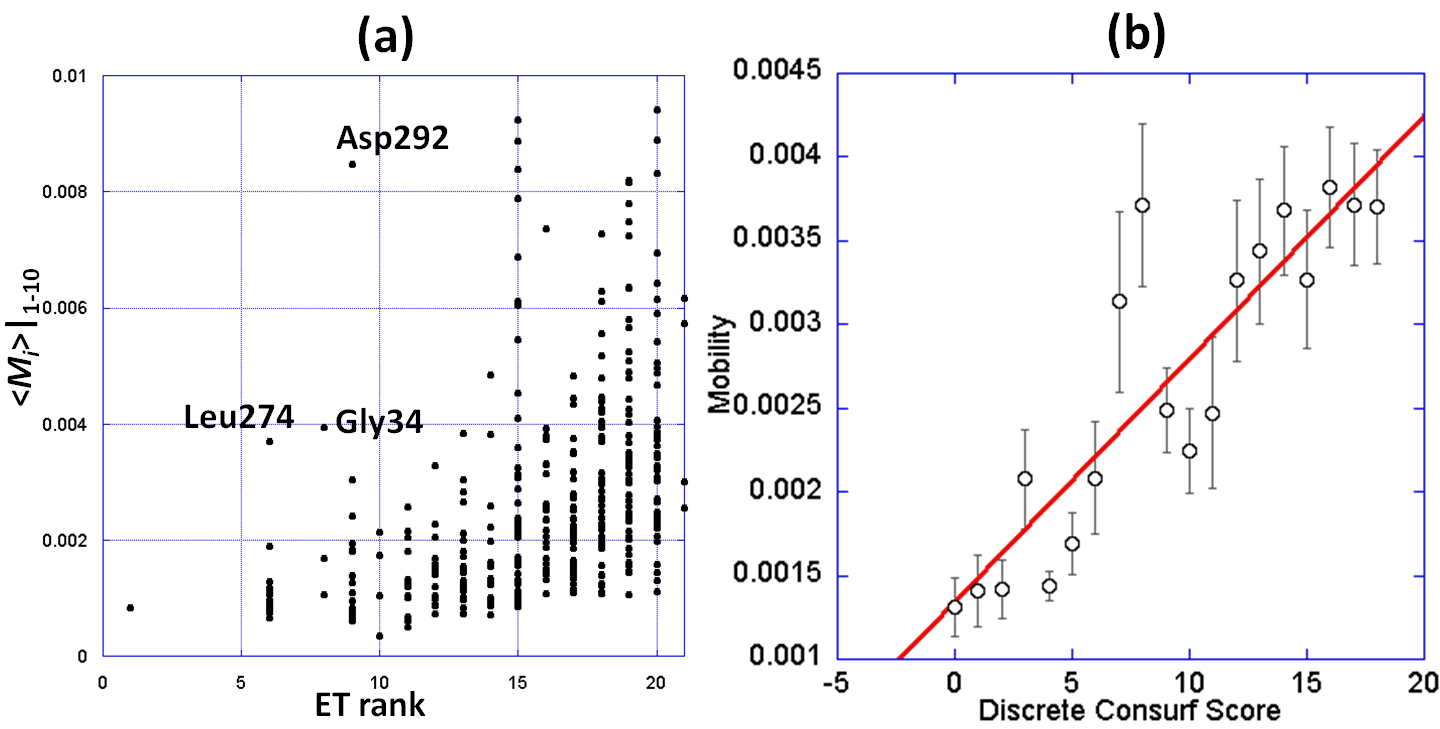

Supplement: Figure S7 — Comparison of residue mobilities with their evolutionary conservation properties. (a) The mobility for each residue averaged ver the first 10 GNM modes is plotted against its ET rank. Three outliers for ET rank 6, 8 and 9 are labeled, two of which (Gly34 and Asp292) are NEF-contacting residues. (b) Proportionality between the discretized ConSurf score and the average mobility (average |1–10) for residues with the same discrete ConSurf score. The discretization is performed by sorting all residues according to the ConSurf score, grouping every 20 consecutive residues and evaluating the mean mobility for each group. The correlation coefficient between average mobility and discrete ConSurf score is 0.88. (0.14 MB PNG) [file pcbi.1000931.s014.png]

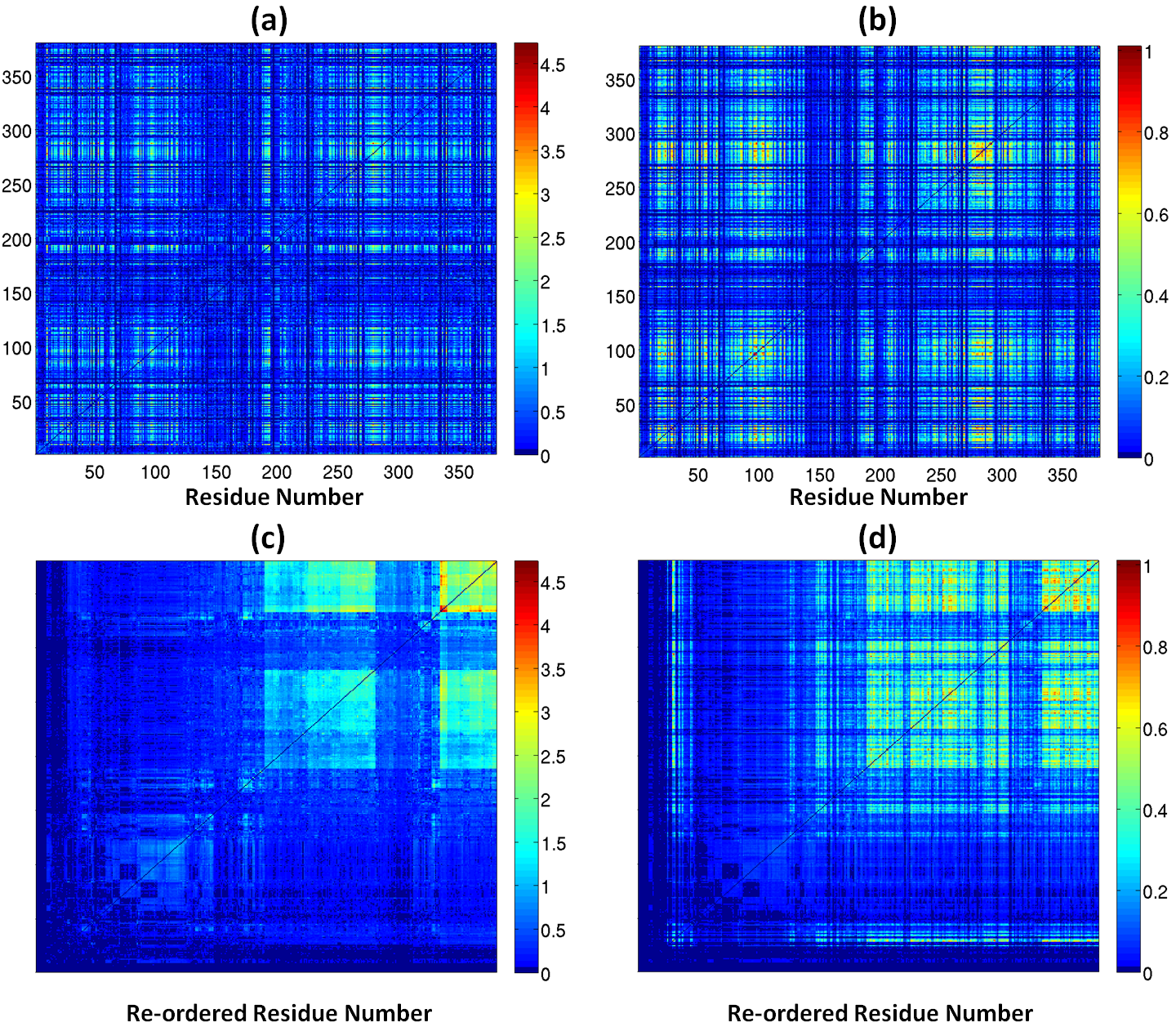

Supplement: Figure S8 — Comparison of correlated mutations map obtained by MI analysis and by the SCA. The first two panels are the correlation maps calculated using (a) SCA and (b) MI. (c) The SCA correlation map after hierarchical clustering (note that the abscissa does not correspond to sequential residues anymore, but those rank-ordered according to their extent of correlated mutations. (d) The MI correlation map with residues re-ordered according to the same permutation in panel (c). (2.18 MB PNG) [file pcbi.1000931.s015.png]

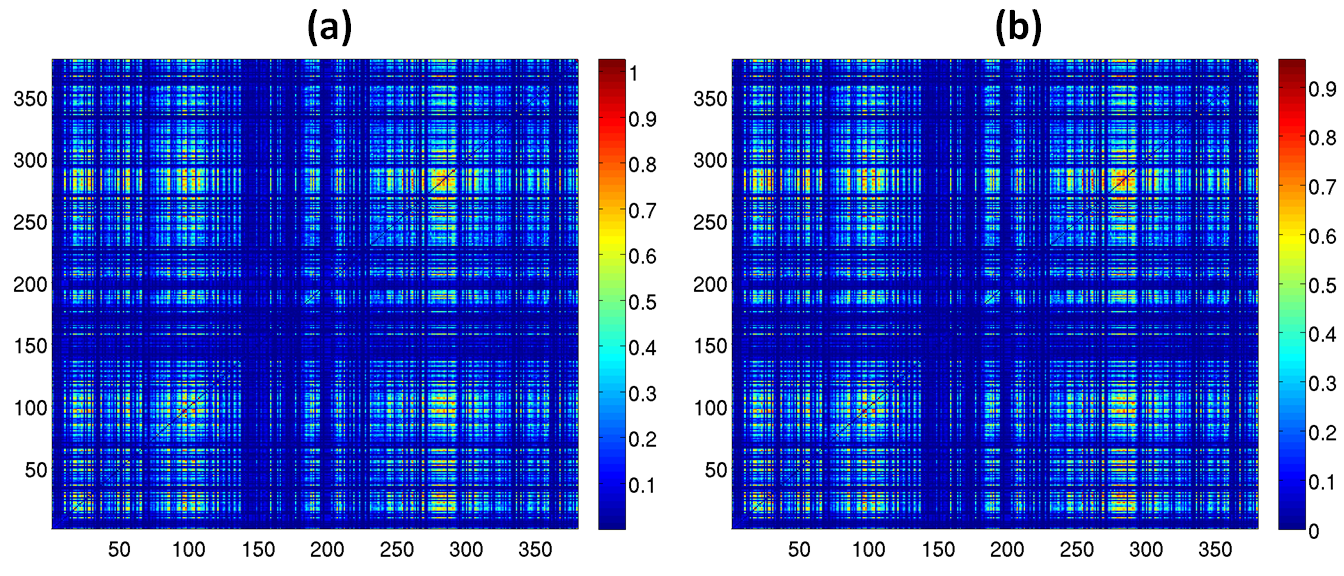

Supplement: Figure S9 — Comparison of MI matrices. Comparison of MI matrices obtained with (a) the original dataset of Hsp70 family sequences (4839 of them), retrieved from Pfam release 22, and (b) the larger dataset retrieved from the Pfam release 24. (1.08 MB PNG) [file pcbi.1000931.s016.png]

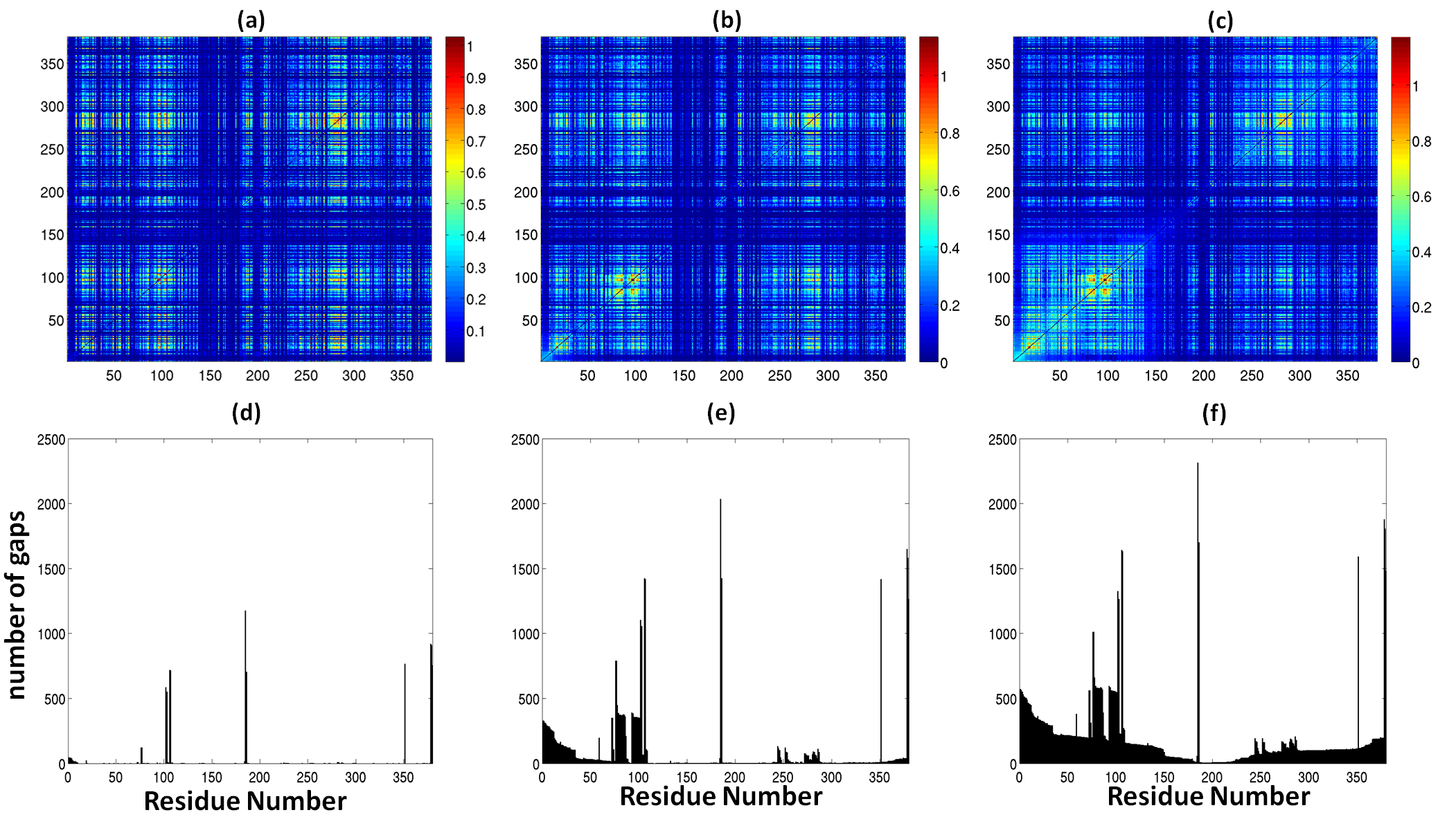

Supplement: Figure S10 — MI Results from MSA with different gap tolerances. The upper panels, (a) (b) and (c), display the MI matrices calculated by allowing sequences with different coverages of the reference sequence, i.e. 2%, 25% and 50% gaps. Panels (d) (e) and (f), show the number of gaps observed in each column of the three respective MSAs. (1.69 MB PNG) [file pcbi.1000931.s017.png]

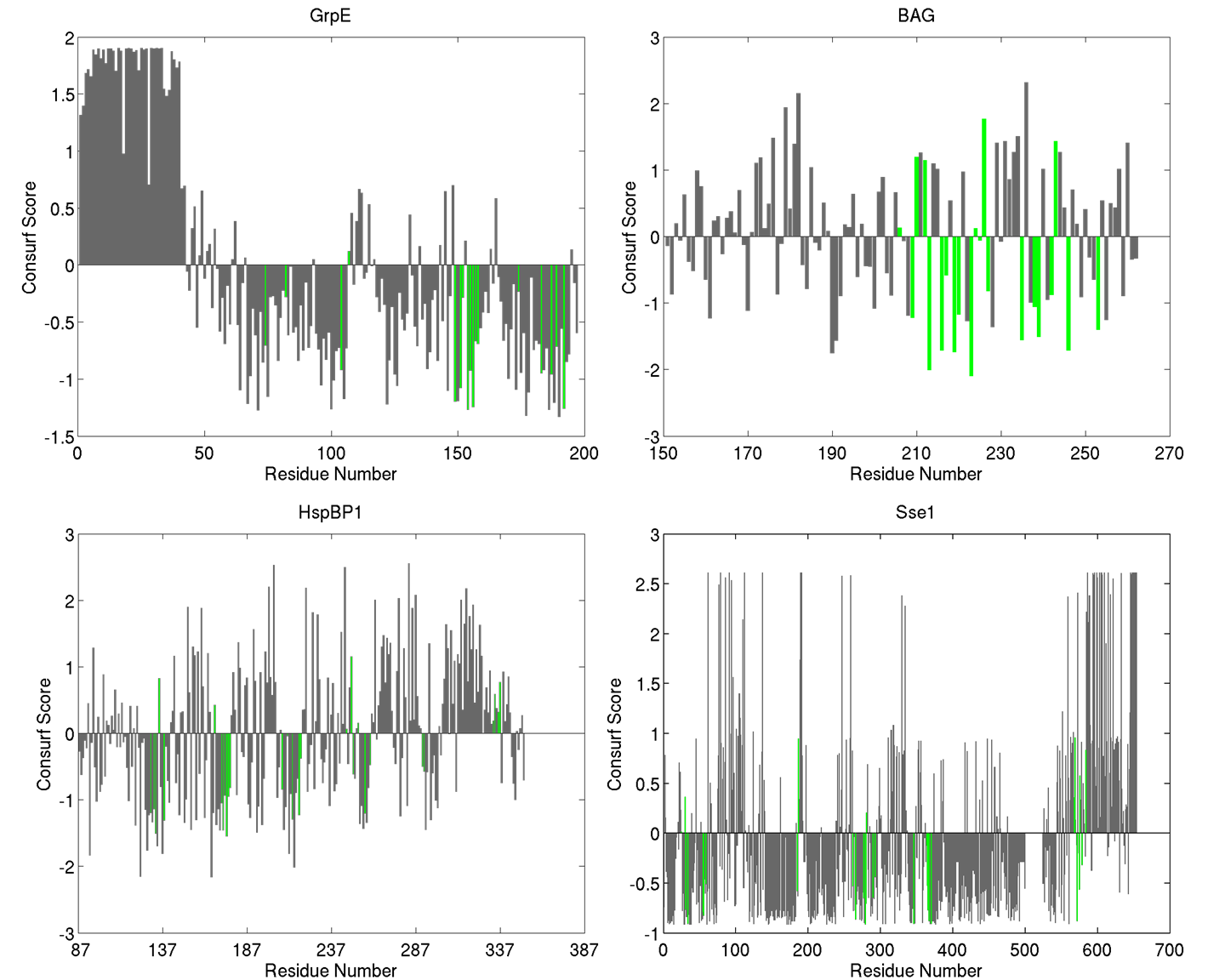

Supplement: Figure S11 — ConSurf score for residues in each NEF. Lower score corresponds to higher conservation. Residues that are in contact with the Hsp70 ATPase domain are colored green. (0.14 MB PNG) [file pcbi.1000931.s018.png]
